# Diagnostic inequalities relating to physical healthcare among people with mental health conditions: a systematic review

**DOI:** 10.1016/j.eclinm.2024.103026

**Published:** 2025-01-10

**Authors:** Elisa Liberati, Sarah Kelly, Annabel Price, Natalie Richards, John Gibson, Annabelle Olsson, Stella Watkins, Emily Smith, Serena Cole, Isla Kuhn, Graham Martin

**Affiliations:** a The Healthcare Improvement Studies (THIS) Institute, University of Cambridge, Cambridge, UK; b Division of Psychology and Language Sciences, University College London, London, UK; c Cambridgeshire and Peterborough NHS Foundation Trust, Cambridge, UK; d Department of Psychology and Human Development, University of East London, London, UK; e School of Clinical Medicine, University of Cambridge, Cambridge, UK; f The McPin Foundation, London, UK

**Keywords:** Diagnosis, Diagnostic inequalities, Diagnostic error, Mental health conditions, Mortality gap

## Abstract

**Background:**

Inaccurate diagnosis of physical health problems in people with mental health conditions may contribute to poorer health outcomes. We review the evidence on whether individuals with mental health conditions are at risk of diagnostic inequalities related to their physical health.

**Methods:**

We searched MEDLINE, PsycINFO, Embase, and CINAHL, 1 September 2002–18 Septemebr 2024 (PROSPERO 2022: CRD42022375892). Seventy-nine studies were eligible for inclusion. Risk of Bias (RoB) was assessed using the Newcastle–Ottawa or RoB2 tools and results were presented as a narrative synthesis.

**Findings:**

Findings from the included studies suggests that people with mental health conditions face diagnostic inequalities for their physical health. A minority of studies adopted a design that specifically measured professional- and service-related factors associated with diagnostic inequalities. Most studies, however, measured diagnostic endpoints only, meaning that no inference could be made regarding the relative impact of patients' and clinicians’ behaviour in producing inequalities.

**Interpretation:**

Further investigations should consider the stage of the diagnostic process at which inequalities occur, to improve knowledge of the mechanisms underpinning diagnostic inequalities, and support the development of targeted improvement interventions.

**Funding:**

This study is funded by The Health Foundation’s grant to the University of Cambridge for The Healthcare Improvement Studies (THIS) Institute. Grant number not applicable.

**Research in context**

**Evidence before this study**

People with severe mental health conditions have a 15–20 year shorter life expectancy than those without. While an evidence base is rapidly developing on the role played by health inequalities in this mortality gap, diagnostic inequalities have not been systematically assessed. We address this gap by systematically reviewing quantitative studies that examined whether individuals with mental health conditions are at risk of having their physical health problems undiagnosed, misdiagnosed or diagnosed late, published between September 2002 and September 2024 and catalogued on four databases (MEDLINE, PsycINFO, Embase, CINAHL).

**Added value of this study**

The pattern of findings from the included studies suggests that people with mental health conditions face diagnostic inequalities for their physical health: of 37 studies with a robust comparator group, 29 found that having one or more mental health conditions is associated with a statistically significant increased risk of having a physical health problem undiagnosed or diagnosed late. A minority of studies (n = 15) used a research design capable of isolating the specific role of healthcare systems in these inequalities: 14 found evidence that people with mental health conditions were at greater risk of diagnostic error than those without. Most studies did not allow inference on the mechanisms underpinning diagnostic inequalities.

**Implications of all the available evidence**

The diagnostic inequalities identified have potentially serious clinical consequences, and tailored improvement actions should be considered. Given the established contribution of professional- and service-related factors, the onus of behavioural change should not be solely on patients. Future research should: consider the stage of the diagnostic process at which inequalities occur; focus on under-represented mental health conditions (personality and eating disorders); and address diagnostic inequalities related to cardiovascular problems, which are the conditions most strongly associated with the mortality gap.

## Introduction

The body of evidence on the excess mortality in people with mental health conditions has developed rapidly over the past twenty years and is now substantial.^1^ In 2024, NHS England declared that people living with severe mental illness (SMI) face “one of the greatest health equality gaps”.^2^ Globally, patients with SMIs have a life expectancy 15–20 years shorter than the general population,^2^^,^^3^ but reduced life expectancy is found across the spectrum of mental health conditions.^4^^,^^5^ Most excess deaths in people with mental health conditions are caused by preventable physical illness^3^^,^^6^, ^7^, ^8^; a 2019 review found that risk of obesity, diabetes, and cardiovascular diseases in this population is 1.4–2.0 times that of the general population.^2^^,^^4^

Some factors are known to contribute to the physical health inequalities affecting people with mental health conditions. These include social and financial disparities, associated poorer lifestyles, the impact of psychotropic medication on physical health, and suboptimal care for physical health problems.^9^, ^10^, ^11^ Inequalities in the diagnosis of physical health problems in this population is also likely to be a problem, but evidence about these has not been systematically assessed.

Diagnostic inequalities are complex, have multiple contributing factors, and may happen at different stages of the diagnostic process^12^, ^13^, ^14^ (Box 1). Diagnostic errors are defined as diagnoses that are missed, incorrect, or delayed, as detected by a subsequent definitive test or finding.^17^ These errors occur after the point of patients' presentation to services, and are usually associated with clinician-related factors (e.g. lack of knowledge or problems in data gathering) and/or system-related factors (e.g. poor care coordination or inefficient processes^13^). One well-known form of diagnostic error is ‘diagnostic overshadowing’, where physical symptoms are misattributed to a mental health condition.^21^

Box 1 **Glossary of diagnostic terms used in this article.**

**Diagnostic inequalities.** Preventable and unwarranted variations in diagnostic processes and outcomes among different population groups.^15^ Diagnostic inequalities may be caused by several factors, individually or in combination, including patient behaviors (e.g. late presentation to services), clinician behaviors or healthcare system issues (diagnostic errors), and broader social inequalities.^15^^,^^16^

Types of diagnostic inequality include:

• **Underdiagnosis**: Some population groups are systematically less likely to receive a warranted diagnosis.
• **Late-stage diagnosis**: Some population groups are systematically more likely to receive a warranted diagnosis at a later stage.
• **Route to diagnosis:** Some population groups are systematically more likely to be diagnosed through specific care routes or pathways (e.g. emergency presentations vs primary care).

**Diagnostic error**. A failure, on the part of a clinician or health service, to establish accurately and in a timely manner the cause of a patient's health issue or communicate the explanation to the patient; such failure is ideally established by a subsequent definitive test or finding.^13^^,^^17^^,^^18^ Types of diagnostic error include:

• **Missed diagnosis**: A type of diagnostic error where a health condition is not diagnosed despite signs and symptoms being reported by the patient.^13^^,^^18^
• **Misdiagnosis**: A type of diagnostic error where a wrong diagnosis is given, and the actual condition goes unrecognised for some time.^13^^,^^18^
• **Delayed diagnosis**: A type of diagnostic error where sufficient data were available to make the correct diagnosis at an earlier point in the course of the diagnostic process.^13^^,^^14^^,^^18^

Due to the complex nature of diagnosis,^14^^,^^19^ diagnostic errors and inequalities are notoriously difficult to measure. However, substantial progress has been recently made in this field.^13^, ^14^, ^15^^,^^17^^,^^18^ For comprehensive visual models of the processes underpinning diagnostic inequalities and how to address them, see the Safer Dx framework^14^ the revised Andersen Model of Total Patient Delay by Walter et al.^20^

Diagnostic errors, however, are not the only contributors to diagnostic inequalities: factors that precede patients' presentation to services may play a part too.^12^ Existing studies have conceptualised the ‘total patient delay’, taking into account factors such as time taken by an individual to identify symptoms and seek medical care.^12^^,^^20^ People with mental health conditions may struggle to seek diagnosis in a timely manner due to the impact of their condition,^22^ financial burdens,^23^, ^24^, ^25^ healthcare models that do not address their needs,^23^, ^24^, ^25^, ^26^ and anticipation of stigmatisation,^27^ of poor-quality care, or of not being taken seriously.^23^, ^24^, ^25^, ^26^^,^^28^

Challenges faced before contact with the healthcare system and those met during the diagnostic process are both important, but they have distinct underlying causes. While it is broadly accepted that individuals with mental health conditions are vulnerable to diagnostic inequalities, an overview of research assessing this problem is lacking. This systematic review addresses this gap.

## Methods

Our primary research question was: what evidence exists that individuals with mental health conditions are at risk of having their physical health problems undiagnosed, misdiagnosed, or diagnosed late? Secondary research objectives included examining which kind of physical and mental health conditions have been examined most frequently; what kind of diagnostic problems have been assessed; and at which point(s) of the diagnostic process these problems occur. Our research aims are deliberately broad to provide a critical assessment of the state of epidemiological knowledge in this area.

The study was conducted and reported following PRISMA guidelines.^29^ The findings are synthesised narratively; no meta-analysis was conducted. A peer researcher (a researcher with personal experience of mental health conditions) contributed to all review stages, including findings interpretation.

### Ethics

This study is a systematic review that synthesises previously published content; ethical approval was therefore not required.

### Search strategy and selection criteria

Database searches were conducted on MEDLINE (Ovid), Embase (Ovid), PsycINFO (EbscoHost), and CINAHL (EbscoHost) on 21/11/2022 (and later updated on 18/09/2024). Additional studies were identified through manual searches.

The search strategy (combining keywords and standardised index terms) was developed using the PICO framework by the authors (including a medical librarian), in collaboration with experts in diagnostic error. Studies in English, published between 01/09/2002 and 18/09/2024, were included. Reviews, letters, editorials, comments, books, book chapters, case studies, and dissertations were excluded. Full search strategies and results are provided in Appendix 1.

Titles and abstracts were independently reviewed by two screeners. We included studies that met the following criteria: primary studies (based on original data collection), using an established quantitative design, providing information about physical health-related diagnostic inequalities in people with a mental health condition. Some conditions, such as dementia and delirium, are at the intersection of mental and physical health; we classified them as mental health conditions, as they include significant psychological symptoms. We included any healthcare setting in any country.

**Fig. 1:**
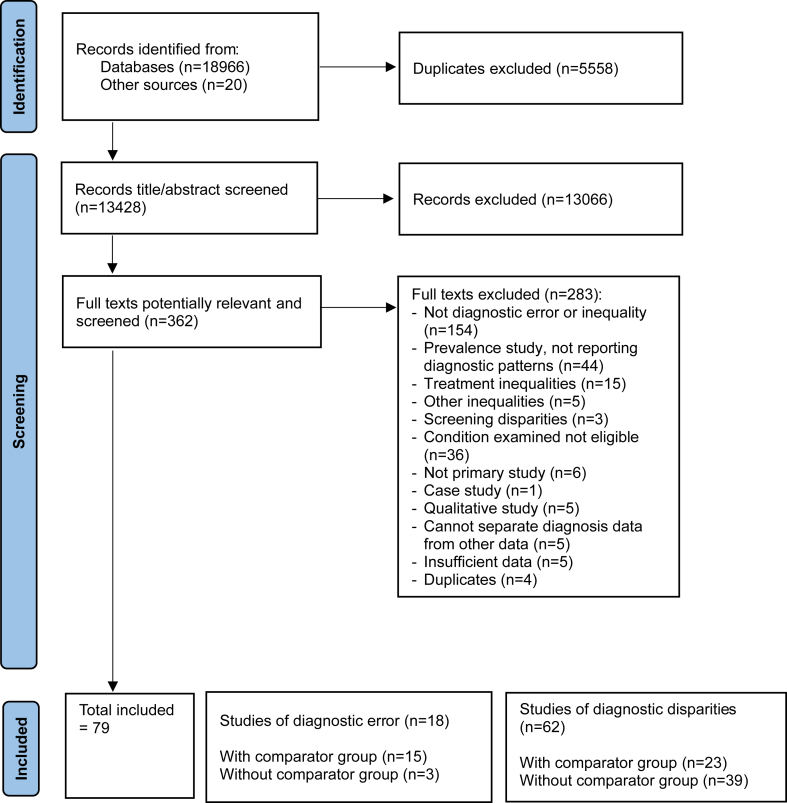
PRISMA chart. Please consider that one of the sources with comparator group (O’Rourke 2008)^30^ separately reports findings relating to both diagnostic error and diagnostic disparities, and is therefore counted in both groups.

We excluded qualitative studies and studies reporting on diagnostic inequalities in mental health conditions (e.g. delayed diagnosis of bipolar) and diagnostic inequalities relating to physical health in those with intellectual or learning disabilities. We also excluded studies on inequalities (such as under-screening, undertreatment, or excess mortality) that did not relate to diagnostic patterns, and studies on incidence and prevalence of physical illness in people with mental health conditions that did not examine diagnostic patterns. The PRISMA chart (Fig. 1) summarises the process and reasons for exclusion.

### Data analysis

Data were extracted by a single author and reviewed by an independent reviewer for accuracy. The data extraction template was piloted and refined on a sample of 10 studies. Risk of Bias (RoB) was assessed by one author using the Newcastle–Ottawa scale for non-randomised studies and with the RoB 2 tool for randomised studies. RoB assessment were solely based on the data relating to diagnostic inequalities, and therefore may not reflect overall study quality. We extracted quantitative measures of diagnostic inequalities (e.g. differences in the duration of diagnostic intervals or in the odds of missed diagnoses between people with and without mental health conditions). We systematically reported outcomes from adjusted models where available, and identify where these were not reported. Given the heterogeneity of study designs, definitions and measurements used, physical and mental health conditions examined, and outcome data, we did not conduct a meta-analysis. We clustered studies based on the kind of diagnostic problems they examined (studies suggestive of diagnostic *error* vs studies indicative of wider diagnostic *inequalities*) and, within these broad categories, examine patterns of physical and mental health conditions targeted. Section 3.2 provides a narrative synthesis of the evidence.

### Role of funding source

This study is funded by The Healthcare Improvement Studies (THIS) Institute, which is supported by the Health Foundation—an independent charity committed to bringing about better health and health care for people in the UK. The views expressed in this publication are those of the authors and not necessarily those of the Health Foundation. The Health Foundation had no role in the writing of the manuscript or the decision to submit it for publication. The authors were not precluded from accessing data in the study, and they accept responsibility to submit for publication.

## Results

### Study characteristics

The search strategy identified 18,966 articles, and 20 were identified through cross-referencing and manual searches. After removing duplicates, the abstracts of 13,428 papers were screened, and 13,066 were excluded. The remaining 362 were full-text reviewed, leading to the exclusion of 283 papers. A total of 79 studies were eligible for inclusion and were data-extracted.

Thirty-seven studies employed a robust mental health comparator group. Only these studies allow for an assessment of the difference in risk of exposure to diagnostic inequalities between people with and without mental health conditions. Our narrative synthesis therefore focuses on these studies. The 42 studies without a robust mental health comparator group are summarised and tabulated in Appendix 2.

Studies differed in the type of diagnostic problems they assessed. Fifteen studies used measures that were suggestive of diagnostic *error:* missed, incorrect, or delayed diagnoses occurring after presentation, meaning that late or non-presentation by patients could be eliminated as a possible cause. Section 3.2.1 and Table 1 describe these studies.

**Table 1:** Studies indicative of diagnostic error.

First author, year	Country	Sample size	Age (y)/sex (M/F)/ethnicity	Study design	Data source/setting	Mental health conditions (clustered), type of analysis (grouped, per-cluster, or per-diagnosis), and diagnostic method	Physical health condition. Diagnosis and diagnostic method	Type and method of assessment of diagnostic issue	Comparator group	Key outcome(s) Odd Ratios (ORs) Hazards Ration (HR) Relative Risk (RR) Adjusted (adj)
**Cancer**										
Benitez Majano 2022	UK	Patients with ‘red-flag’ signs of colon cancer) = 2115. With mental health conditions = 308. Without mental health condition = 1807	Median 75 (IQR 65–82)/M (with mental health condition) = 37.9%. M (without mental health conditions) = 51.5%/ethnicity N/A	Retrospective cohort	Linked primary care data from practices in England with cancer registry and hospital data	• **Mood disorders** (depression, anxiety) 92% • **Psychotic disorders** (bipolar) • **Substance misuse** (alcohol addiction) • **Eating disorders** (anorexia and bulimia) Grouped analysis Database read codes and prescriptions.	Colon cancer (ICD-10 codes)	DELAYED DIAGNOSIS Assessed length of intervals to cancer diagnosis in patients with psychiatric conditions who presented with symptoms of as-yet–undiagnosed colon cancer.	Without mental health conditions	In quantile regression, after accounting for co-variables, the diagnostic interval (consultation-to-diagnosis) of those with mental health conditions vs those without was 466 days (95% CI 413–519 days) vs 365 days (95% CI 288.6–442.4) at the 75th centile, p < 0.001; and 224 days (95% CI 159–290 days) vs 126 days (95% CI 94.5–157.5) at the 50th centile, p = 0.003.
Mounce 2017	UK	Patients with colorectal cancer and comorbid conditions = 4512. Not reported for anxiety/depression subgroup	Adults >/ = 40 y (age is reported in seven bands)/M 54.0%/ethnicity N/A	Retrospective cohort	Electronic primary care records	• **Mood disorders** (depression, anxiety) Per-cluster analysis Database read codes	Colorectal cancer (database read codes)	DELAYED DIAGNOSIS Assessed time from first symptomatic presentation (first entry of a code for colorectal cancer) to diagnosis of cancer.	Without anxiety or depression (as reference group)	Regression model found that anxiety/depression was associated with longer diagnostic intervals for colorectal cancer: 9-day diagnostic delay (95% CI 3–17), coeff. (95% CI) 0.11 (0.03, 0.20), p = 0.007. (Model adjusted for age and gender).
Van Hout 2011	The Netherlands	Total = 197. Subgroup with mental health conditions = 11 (5.6%).	Mean (SD): 68.73 (12.07)/M 50.3%/ethnicity N/A	Retrospective cohort	Routine care data of the Primary Care Network Utrecht, a network of 23 general practices in the Netherlands	• **Mood disorders** (depression, anxiety) Per-cluster analysis Primary care database	Colorectal cancer	DELAYED DIAGNOSIS Assess time between first consultation with general practitioner and referral to specialist or endoscopy unit (T2); and time between referral and histological diagnosis (T3).	Without anxiety or depression	In univariate analysis, anxiety and depression were significantly associated with delay at T2: OR (95% CI): 3.87 (1.13–13.30), but not at T3. In multivariate analyses, the association between psychiatric comorbidity and T2 delay remained: adj OR 3.97 (1.14–13.85).
Walter 2016	UK	Total = 2507 in overall cohort. Not reported for anxiety/depression sub-group.	Adults>/ = 40 Median 65 (range 40–100)/47.1% M; 52.9% F/98.1% white	Retrospective cohort	Secondary care hospitals (n = 4) with primary care data, hospital data and validated questionnaire	• **Mood disorders** (depression, anxiety) Per-cluster analysis Diagnostic method not stated explicitly but appears to be patient questionnaire and primary care data.	Colorectal cancer (first confirmatory histology report (ICD codes C18–C20) or first clinical diagnosis in hospital medical record).	DELAYED DIAGNOSIS Reports ‘Health Service Interval’ (HSI), defined as the time from first presentation to diagnosis. (Also reports Total Diagnostic Interval (TDI)—time from first symptom onset reported by patients to diagnosis).	Without anxiety or depression (as reference group)	In multivariable analysis, people with anxiety/depression had a longer interval from presentation to diagnosis (measured as HR). They were diagnosed 0.8 (0.71–0.90, 95% CI) times as quickly as those without anxiety/depression; p < 0.001. Anxiety/depression was also associated with a longer overall diagnostic process. HR for time from first symptom onset to diagnosis: 0.86 (0.77–0.96); p < 0.001.
Iglay 2017	US	Total = 16,636. Subgroup with mental health conditions = 3961	Women >/ = 68. With mental health conditions = 68–64 y (41.7%); 75–84 y (46.7%); 85+ y (11.7%)/Female: 100%/White: 90.25%. African American: 5.6% (5.9%).	Retrospective cohort	Medicare data (linked Surveillance, Epidemiology and End Results data (SEER))	• **Mood disorders** (depression, anxiety) • **Psychotic disorders** (bipolar, schizophrenia, and other psychotic disorders) Per-cluster analysis ICD-9 codes	Breast cancer (early stage I-IIIa); (Medicare codes).	DELAYED DIAGNOSIS Time interval from date of first Medicare claim for breast symptoms to breast cancer diagnosis.	Without anxiety, depression, or severe mental illness.	Patients with comorbid anxiety and depression had an 11% increased risk for diagnosis delay of ≥ 90 days from symptom recognition to diagnosis (adj RR = 1.11; 95% CI 1.00, 1.23). *No significant differences were found for other mental health clusters, or for patients with any mental health for overall risk of diagnosis delay at 60 days.*
O'Rourke 2008	US	Total = 160. Subgroup with mental health conditions = 52. Subgroup without mental health conditions = 108.	Mean (SD). No mental health conditions: 65.8 (10); with mental health conditions: 64.6 (11.2)/M 87.5/Caucasian 98%	Retrospective cohort	Veteran's Administration Hospital Data	• **Mood disorders** (major depression [79% of the sample], PTSD) • **Psychotic disorders** • **Cognitive** (schizophrenia) disorders (dementia) • **Personality disorders** Grouped analysis DSM-IV	Oesophageal cancer (ICD-9 code)	DELAYEDDIAGNOSIS Time from onset of alarm symptoms (reported by patients in the initial intake history) to diagnosis (Stage of cancer at diagnosis is reported in Table 2).	Without mental health conditions	Patients with psychiatric illness had a longer interval from onset of alarm symptoms to oesophageal cancer diagnosis compared to patients without psychiatric illness: median 90 (IQR 20–162) days vs 35 (IQR 0–76) days, p = 0.001. Multivariate analysis showed that psychiatric illness in general, and specifically depression, were predictive of delayed diagnosis. Psychiatric illness, adj HR = 0.605 (0.424–0.862); depression, adj HR = 0.622 (0.425–0.910). (HR < 1 indicates a lower ‘hazard’ for being diagnosed and therefore a longer time to diagnosis).
Iachina 2017	Denmark	Sample with depression = 508. Control (no depression) = 27,234.	Adults >/ = 18 y/M with depression: 37.8%; no depression: 51.8%/ethnicity N/A	Retrospective cohort	Danish national registries and databases (n = 4)	• **Mood disorders** (depression) Per-diagnosis analysis Hospital contact due to depression (depressive episode/recurrent depressive disorder) within 10 years before primary lung cancer diagnosis. ICD-10 codes.	Lung cancer (non-small cell); (ICD-10 codes).	DELAYED DIAGNOSIS Duration of the diagnostic process: days of primary investigation; stage at diagnosis.	Without depression (no hospital contact for depression)	*Multivariate analysis showed no difference in the duration of the diagnostic process between those with and without depression (adj HR = 0.99; 95% confidence interval 0.90; 1.09).*
**Cardiovascular illness**										
Sharp 2022	US	Total (acute myocardial infarction hospital encounters) = 44,473. Subgroup with mental health conditions = 10,593 (23.8%). Myocardial infarction diagnoses missed = 574	Adults. Mean: Cases 67.9 (14.0); controls: 68.9 (14.2)/F 57.2%/White 38.5%; Hispanic 37.0%; Black 13.4%; Asian/Pacific Islander 7.3%	Retrospective cohort	Data from a single integrated health system; Emergency department data.	• **Mood disorders** (anxiety, others unspecified) • **Psychotic disorders** (schizophrenia-related disorders) • **Substance misuse** (alcohol-related and others) Per-cluster analysis ICD codes	Acute myocardial infarction; (ICD-9 and ICD-10 codes).	MISSED DIAGNOSIS In look-back analysis, patients with an emergency department diagnosis of nonspecific chest pain or dyspnoea in the 30 days prior to an acute myocardial infarction hospitalisation were considered a ‘missed acute myocardial infarction case'.	Without mental health conditions and substance misuse (as reference)	Adjusted OR of missed acute myocardial infarctions diagnoses were higher in those with mental health conditions than those without: (adj OR 1.48, 95% CI 1.23–1.77) and for those with mental health diagnoses and substance misuse compared with those with neither disorder (adj. OR 1.90, 95% CI 1.30–2.76). Adj OR for patients with substance misuse was not statistically significant (OR 1.22, 95% CI 0.91 to 1.62). In per-diagnosis analysis, patients with anxiety or other mood disorders had higher proportions of missed acute myocardial infarctions (anxiety: 1.9% vs 1.2%; difference in proportions 0.7%; 95% CI 0.4–1.1; mood disorders: 1.8% vs 1.2%; difference in proportions 0.6%; 95% CI 0.3–1.0)
Waxman 2018	US	Total = 1,561,940 participants; (Of these overall cases were: depression 1,064,088; dementia 319,139 but these conditions may have co-existed)	Mean (SD) 77.9 (10.3)/54.1% F/Across the 5 conditions: White: 77.4–90.7%; Black: 4.8–12.9%; Asian/Pacific Islander: 1.3–3.1%; Hispanic 2.2–7.0%	Retrospective cohort	Medicare claims. ED department	• **Mood disorders** (depression) • **Cognitive disorders** (dementia) Per-diagnosis analysis	5 conditions: Ruptured Aortic aneurysm, acute MI, stroke, aortic dissection, and subarachnoid haemorrhage	MISSED DIAGNOSIS To estimate proportions of emergency department visits which showed symptoms of acute vascular incidents and ended in discharge without a diagnosis (observed vs expected). To identify patient characteristics independently associated with missed diagnostic opportunities.	Without depression and dementia (as reference)	Excess emergency department discharges (one or more emergency department discharges within the 45 days preceding the index hospital admission) were positively correlated with having dementia and depression (among a range of other health conditions). Adj OR for depression for all 5 conditions was 95% CI >1; for dementia all >1 apart from aortic dissection.
Byrd 2012	US	Total = 168,630. Anxiety = 3531, depression = 10,455. Control (no anxiety/depression) = 150,905	Mean (95% CI): 51.9 (51.8, 51.9)/52% F/Black: 6%; Hispanic 9%; White 52%; Unknown: 25%; Other 7%.	Retrospective cohort	Integrated healthcare delivery systems. Data from the Cardiovascular Research Network Hypertension Registry	• **Mood disorders** (depression, anxiety) Per-cluster analysis ICD-9	Hypertension	MISSED and DELAYED DIAGNOSIS Missed diagnosis: a diagnosis of hypertension (or prescription of hypertension treatment) has not occurred within 1 year after the second elevated blood pressure instance. Delayed diagnosis: time from second elevated blood pressure to the receipt of a diagnosis of hypertension, or receipt of an antihypertensive medication.	Without depression or anxiety	In multivariable analysis, *the probability of receiving a diagnosis of hypertension by 12 months after the second elevated blood pressure was not significantly different in patients with anxiety and depression than in patients without these diagnoses* (adj HR for anxiety and depression 0.94, 95% CI 0.89–1.00), but it was lower for those with anxiety alone (adj HR 0.93, 95% CI 0.88–0.99) and depression alone (adj HR 0.93, 95% CI 0.90–0.97) compared with patients with neither condition. Median days to diagnosis after the second elevated blood pressure incident was longer in patients with depression and anxiety compared with patients without these diagnoses (31 days, IQR 0–174 vs 5 days, IQR 0–126 days; p < 0.001).
**Other physical health conditions**										
Barin 2020	Switzerland	Multiple sclerosis patients = 522. Subgroup with depression n = 53 (23.5%)	Median 47 (range 38–54) at baseline/F 73.6%/ethnicity N/A	Retrospective cohort	Swiss Multiple Sclerosis registry.	• **Mood disorders** (Depression as a first symptom). Per-diagnosis analysis Self-reported but likely to have been confirmed by a clinician.	Multiple Sclerosis (confirmed by treating physician)	DELAYED DIAGNOSIS Time from evaluation (first visit with a physician) to diagnosis. Logistic regression model with evaluation-to-diagnosis duration as outcome variable (</ = 6 months, >6 months).	Without depression (as reference)	In multivariate analysis, association between depression (as a concomitant first symptom) and prolonged time from specialist evaluation to diagnosis (comparing time </ = 6 months to time >6 months): adj OR 0.46 (0.24, 0.91). This indicates greater likelihood of longer time from symptom evaluation to diagnosis. Proportion of people with time from symptom evaluation to diagnosis </ = 6 months: 9% vs >6 months: 15%.
Nassery 2021	US	Total hospitalizations = 171,666. Principal diagnosis of sepsis = 3468. Treat-and-release emergency department encounter in the 31 days prior to the sepsis admission = 766 (22%). Altered mental status = 33.	Mean: 66.4 (16.0)/F 48.3%/White 37.8%; Black 41.6%; Asian/Pacific Islander 7.4%	Retrospective cohort	Data from a single integrated health system; Emergency department data.	• **Cognitive disorders** (Altered mental status [AMS]). Per-diagnosis analysis ICD diagnosis codes associated with the ED encounters and grouped them using the standard Healthcare Cost and Utilization Project (H-CUP) Clinical Classifications Software (CCS))	Sepsis (ICD 9 and 10 codes)	MISSED DIAGNOSIS Examines hospitalisations for sepsis associated with a prior treat-and-release encounter in the emergency department to identify antecedents of sepsis missed diagnosis. Altered mental status was one condition examined.	No altered mental status (as reference)	Comparing the observed and expected (O:E) rates of sepsis, authors found that altered mental status was one of the two strongest predictors of downstream sepsis hospitalisation after a treat-and-release episode (O:E 2.86 95% CI 2.04–4.00), along fluid and electrolyte disorders. (Not clear whether model is adjusted).
Fernholm 2020	Sweden	Total = 4536. Controls = 44,949 controls.	Cases: 49 (SD 21); Controls: 49 (SD 21) 57%/Cases: F 57%; Controls: F/ethnicity N/A	Retrospective case–control	Data from primary health care and emergency depts. (nationwide databases of patient-reported harm/safety incidents in health care facilities)	• **Mood disorders** (depression, anxiety [N = 160]) • **Psychotic disorders** (n = 20) • **Substance misuse** (alcohol- and drug-related, n = 104), • **Cognitive disorders** (dementia, n = 27) Grouped analysis Diagnosis recorded during the 3 years preceding the preventable harm using ICD-10 codes	Preventable harm (somatic), of which diagnostic error were a subtype. (ICD-10 codes)	DIAGNOSTIC ERROR (UNSPECIFIED) Review of all cases of reportable, preventable harm. Instances of diagnostic error were assessed and confirmed by medically trained staff.	Without mental health conditions	After adjusting for income and education, patients with psychiatric diagnoses had a nearly two-fold higher risk of being subject to preventable harm (adj OR), 1.96; (p < 0.001); 1.69. Of all reported cases of preventable harm, 46% involved diagnostic error of somatic disease.
**Experimental, vignette-based design**										
Isbell 2023	US	Total physicians = 59; n = 27 exposed to depression vignette; n = 32 exposed to control (no depression). (n = 159 randomised but this included non-physicians who were later excluded)	Mean age (physicians): depression 52.07 (14.05); control 50.26 (14.29)/Gender: depression 85.2% M, 14.8% F. Control 81.2% M, 18.8% F/Ethnicity depression: 77.8% White, 11.1% Asian, 0% Black. Control: 81.3% White; 9.4% Asian; 3.1% Black.	Experimental design (randomised)	Physicians recruited from online lists of medical practitioners.	• **Mood disorders** (depression) Per-diagnosis analysis Medical records	Pernicious anaemia	MISSED DIAGNOSIS or WRONG DIAGNOSIS Physicians were presented with a vignette describing a patient with a complex presentation of pernicious anaemia and randomised to diagnose a patient with or without comorbid depression. Diagnostic accuracy (presence or absence of correct diagnosis) was scored by two highly experienced physicians.	Without comorbid depression	Diagnostic accuracy by the physicians was lower in the depression compared to control condition (59.4% vs 40.7%; p = 0.15). Diagnostic accuracy was related to the number of tests ordered by physicians. Accuracy was lower in the depression condition (vs control) when physicians ordered fewer tests (1 SD below mean; OR = 0.103, p = 0.028) but there was no difference when physicians ordered more tests (1 SD above mean; OR = 2.042, p = 0.396).
McDonald 2003	US	Total nurses = 60 (each responding to a vignette describing a man with possible myocardial infarction (MI).	Nurses (n = 60) mean age: 37.7 (SD 7.8). Gender (n = 60): 93.3% F Ethnicity (n = 60): 81.7% white	Experimental design (randomised)	Clinical setting. Nurses were told the study was about clinical decision-making (used Clinical Decision Making Instrument) rather than unconscious stereotyping.	• **Psychotic disorders** • **Mood disorders** (anxiety) Per-cluster analysis. Psychosis (nurses read a vignette describing patient on medications for schizophrenia). Anxiety (nurses read a vignette describing patient on anti-anxiety medication).	Myocardial infarction (MI)	MISSED DIAGNOSIS Nurses were randomly assigned to 3 groups: psychosis, anxiety, and control. All groups read a vignette describing a man with possible myocardial infarction (MI) and were asked to respond to these symptoms. The psychosis group also read the man was on schizophrenia medication. The non-psychotic group also read the man was on anti-anxiety medication.	Control group: read a vignette describing a patient without medication for schizophrenia or anxiety.	Significant differences emerged across the three groups in nurses' estimated probability that the patient was experiencing an MI (p < 0.05). Nurses in the psychosis group estimated a mean probability of 35.0% that the patient was having an MI (SD 18.2) whereas nurses in the nonpsychotic condition (control group) predicted 50.6% probability (SD 28.2). Nurses in the anxiety group: 49.5%. (SD 19.3).

**Fig. 2:**
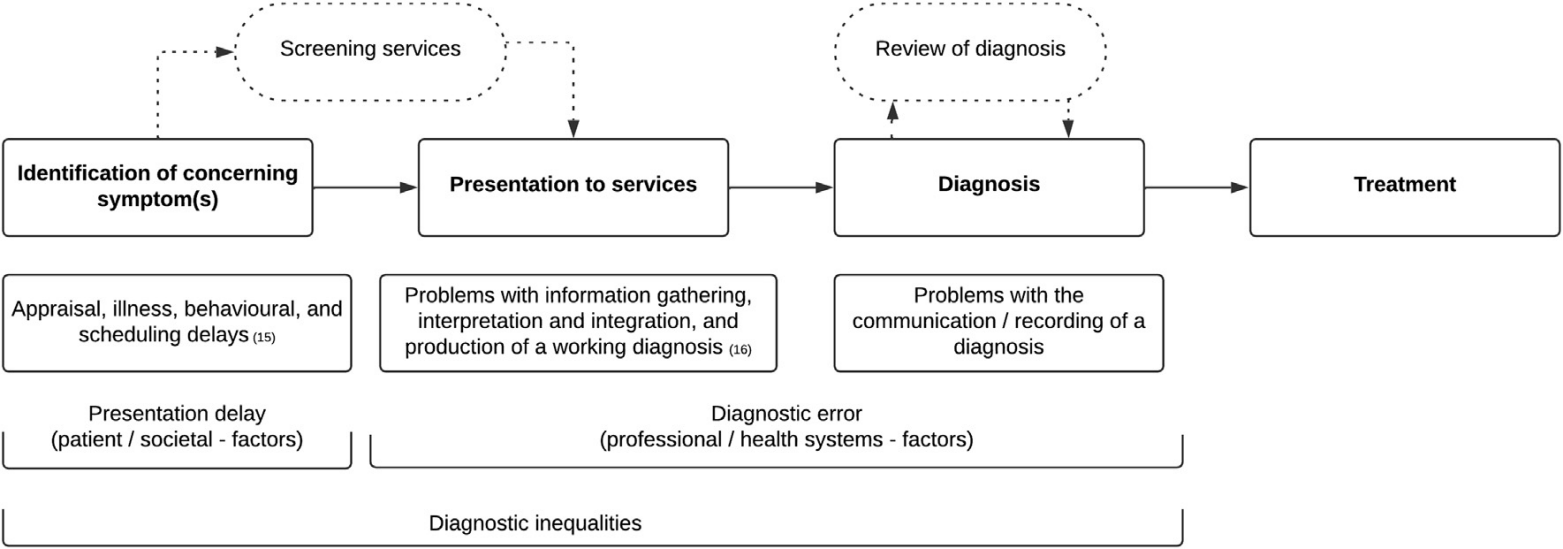
Components of the diagnostic process (adapted from Andersen et al., 2005^12^ and the National Academies of Science, Medicine and Engineering report Improving Diagnosis in Health Care^13^).

Twenty-three studies examined diagnostic *inequalities*: their design meant it was not possible to distinguish between problems relating to the development of a diagnosis by health professionals (diagnostic error) and problems relating to patients' late or non-presentation to services. In this group of studies, which we name ‘wider diagnostic inequalities’ (Section 3.2.2, Table 2), patterns of inequalities may result from factors relating to diagnostic error, patients' late or non-presentation, or both (Fig. 2).

**Table 2:** Studies of wider diagnostic inequalities.

First author, year	Country	Study design	Data source/setting	Sample size	Mental health conditions (clustered), type of analysis (grouped, per-cluster, or per-diagnosis), and diagnostic method	Physical health condition (diagnosis methods)	Method of assessment of diagnostic disparities	Comparator group	Key outcome(s) Odd Ratios (ORs) Hazards Ration (HR) Adjusted (adj)
**Cancer**									
Fleming 2005	US	Retrospective cohort	National cancer institute (NCI) data	N = 17,468	**Various psychiatric conditions** (unspecified) Grouped analysis	Breast cancer	LATE-STAGE DIAGNOSIS Association between psychiatric condition and stage of cancer at diagnosis	Without psychiatric condition	Risk of late-stage diagnosis of breast cancer: women with psychiatric conditions: OR 1.25 (1.14–1.36) significantly more likely to be diagnosed at an advanced stage. The association remained significant in multivariate analysis. Women with psychiatric conditions had a 20% increased odds of being diagnosed with late-stage disease.
Goodwin 2004	US	Retrospective cohort	Data from the SEER tumour registry and linked Medicare claims data	Total (women with breast cancer) = 24,696. With depression = 1841.	• **Mood disorders** (depression) Per-diagnosis analysis ICD-9-CM	Breast cancer (SEER Medicare claims data). Tumour size obtained from SEER data, stage at diagnosis measured using AJCC staging classification.	LATE-STAGE DIAGNOSIS Association between prior diagnosis of depression and tumour size and stage.	Without depression	*NO SIGNIFICANT ASSOCIATION* *No difference in stage at diagnosis and tumour size between depressed and non-depressed women.* However, in one multivariate analysis, controlling for total number of physician visits in the year before breast cancer diagnosis, depression was associated with later-stage diagnosis (OR 1.25, 95% CI 1.11–1.41 for each increase in AJCC stage) and increased tumour size (OR 1.31, 95% CI 1.16–1.47 for each 10 mm increase in size).
Cunningham 2015	New Zealand	Retrospective cohort	New Zealand Cancer Registry; Mental Health Information National Collection; Project for Integration of Mental Health Data	Total sample n = 8772. Patients with functional psychosis = 112. Patients with any other psychiatric diagnosis or no psychiatric diagnosis = 328. People with no recorded contact with mental health services = 8322	• **Psychotic disorders** (functional psychosis: schizophrenia, schizoaffective disorder, or bipolar disorder). Per-cluster analysis ICD10 codes.	Breast or colorectal cancer	LATE-STAGE DIAGNOSIS Examined cancer-specific survival for patients with and without psychosis and measured the impact of late-stage diagnosis on this.	Without contact with mental health services	After adjusting for age and ethnicity, those who had been diagnosed with functional psychosis prior to cancer diagnosis had 2.5–3 times higher risk of death within 5 years compared to comparator group (breast cancer: adj HR 2.55 (95% CI 1.49–4.35); colorectal cancer: adj HR 2.92 (95% CI 1.75–4.87)). Late stage at diagnosis explained more than a third of the survival difference for people with functional psychosis.
Cespedes 2020	Spain	Retrospective case–control	Medical records	Total sample = 111 With mental health conditions = 37. Without mental health conditions = 74	• **Mood disorders** (unspecified) • **Psychotic disorders** (schizophrenia spectrum, bipolar) Per-cluster analysis ICD-9 codes	Breast or colorectal cancer	LATE-STAGE DIAGNOSIS Association between having a mental health condition and risk of diagnosis at advanced cancer stage (clinical stages IIIA, IIIB, IIIC, and IV).	Without mental health conditions	People with mental health conditions have higher risk of advanced cancer stage diagnosis compared to people without mental disorders: OR 3.93 (1.6–9.65, p = 0.002). Multivariate analysis identified variables associated with cancer stage at diagnosis to be previous severe mental disorder (adj OR, 4.67; 95% CI, 1.73–12.61) and older age at the time of detection of the cancer. No statistically significant differences were found on correlating cancer stage at diagnosis between different mental disorders groups (p = 0.47; *χ*^2^ test).
O'Rourke 2008	US.	Retrospective cohort	Veteran's Administration Hospital Data	Total sample = 160 With psychiatric conditions = 52. Without psychiatric conditions = 108.	• **Mood disorders** (depression (79%), anxiety, PTSD) • **Cognitive disorders** (dementia) • **Psychotic disorders** (schizophrenia) • **Personality disorders** Grouped analysis DSM-IV	Oesophageal cancer (ICD-9 code)	LATE-STAGE DIAGNOSIS Association between having a mental health condition and cancer stage at diagnosis.	Without mental health conditions	In multivariate analysis, patients with psychiatric illness presented more often with metastatic disease than those without (37% vs 18%; p = 0.009). (Delayed diagnosis analysis reported in Table 1)
Chang 2013	UK	Retrospective cohort	Medical records. People using secondary mental health services and severe mental illness.	Total sample = 28,477. Received secondary mental healthcare = 2206	• **Mood disorders** (depression, anxiety disorders) • **Psychotic disorders** (schizophrenia, bipolar disorder, schizoaffective disorder) • **Substance misuse** • **Personality disorders** Per-cluster analysis ICD codes	Cancer	LATE-STAGE DIAGNOSIS Measured the risk of advanced stage cancer at diagnosis for those with mental health conditions.	Without the considered psychiatric diagnosis, and in the same residence area	*NO SIGNIFICANT ASSOCIATION was found between individual mental health conditions and risk of advanced stage diagnosis (OR were not significant in adjusted model). However, many of the mental health condition groups had worse subsequent survival (severe mental illness as a cluster, schizophrenia and schizoaffective disorder individually, and depression, dementia and substance use disorders prior to the cancer diagnosis).*
Lin 2016	US	Retrospective cohort	Department of Defence's Central Cancer Registry and the US Military Health System Data Repository.	Patients with pre-existing mental health condition = 1858.	• **Psychotic disorders** • **Cognitive disorders** (dementia) • Mood disorders (anxiety) • **Substance misuse** • Other (‘any mental health disorder’) Per-cluster analysis ICD-9 or CPT codes	Primary non-small cell lung cancer.	LATE-STAGE DIAGNOSIS Examined the association between a pre-existing mental health condition and: 1) cancer stage at diagnosis, 2) receipt of cancer treatments, and 3) all-cause mortality.	Without a pre-existing mental health condition.	*NO SIGNIFICANT ASSOCIATION. No statistically significant difference in disease-stage at diagnosis between people with and without mental health conditions. For those with any pre-existing mental health condition, adj ORs (95% CI) for diagnosis at late-stage of cancer compared to diagnosis at early stage of cancer was 0.95 (0.83–1.08) compared to OR 1.00 (as reference standard) for those without mental health conditions. No significant ORs were observed between specific mental health disorders and tumour late stage. However, patients with a mental health disorder had a higher mortality than those without (adj HR = 1.11, 95% CI = 1.03–1.20).*
Farasatpour 2013	US	Retrospective cohort	Department of Veterans Affairs (DVA) system (34 facilities)	N = 56, control group N = 478	• **Psychotic disorders** (schizophrenia or schizoaffective disorder) Grouped analysis • ICD-9	Breast cancer	LATE-STAGE DIAGNOSIS Compares between patients with and without schizophrenia for time of presentation	Without schizophrenia or schizoaffective disorder	Presence of large cancerous breast masses at diagnosis: of the 41 patients with schizophrenia/schizoaffective disorder and for whom the size of the breast mass was known, 27 (66%) had large masses (>2 cm) compared with 44% in the control group. Presence of metastatic breast cancer at diagnosis: 12 of the 56 patients with schizophrenia/schizoaffective disorder (21%) had metastatic breast cancer at diagnosis, compared with 20 of 478 in the control group (4%). Authors conclude that patients with schizophrenia often have advanced-stage disease at diagnosis. (Bivariate analysis. No significance data were reported.)
Cunningham 2024	New Zealand	Retrospective cohort	National mental health service use datasets (2002–2018), linked to national cancer registry and hospitalisation data (2006–2018). Study population age 15+.	Contact/no contact with mental health services: Lung cancer: 22,958/1125; prostate cancer: 37,323/794; breast cancer: 3404/1442; colorectal cancer: 33,615/1027.	• **Various psychiatric conditions** (3 or more contacts with mental health or addition services) within the five years before cancer diagnosis • **Psychotic disorders** (bipolar, schizophrenia or psychotic disorders) Grouped and per-cluster analysis ICD-10 codes for psychotic disorders	Lung, prostate, breast or colorectal cancer; (ICD-10) diagnosis codes)	ROUTE TO DIAGNOSIS Emergency presentation (hospital admission within 30 days of cancer diagnosis)–as an indicator of missed opportunities for early diagnosis of cancer.	No contact with mental health services.	For all four cancers, rates of emergency presentation were significantly higher in people with a history of mental health or addiction service use than people without (lung cancer, (rate ratio) adj RR 1.19, 95% CI 1.13, 1.24; prostate cancer adj RR 1.69, 95% CI 1.44, 1.93; breast cancer adj RR 1.42, 95% CI 1.14, 1.69; colorectal cancer adj RR 1.31, 95% CI 1.22, 1.39). Rates were even higher in the subgroup of patients with psychotic disorders (lung cancer, (rate ratio) adj RR 1.23 (1.12, 1.33); prostate cancer adj RR 2.03 (1.41, 2.65); breast cancer adj RR 1.70 (1.16, 2.24); colorectal cancer adj RR 1.35 (1.18, 1.52). Fully adjusted models: for age, sex (for lung and colorectal cancers), ethnicity, area deprivation and stage at diagnosis.
Virgilsen 2022	Denmark	Retrospective cohort	Register data on hospital contacts and prescription medication.	Patients with cancer = 155,851 with. Subgroup of patients with psychiatric conditions = 32,255.	• **Mood disorders** (anxiety) • **Substance misuse** • **Psychotic disorders** (schizophrenia, psychosis) • Other (organic disorders). Per-cluster analysis. ICD-10 codes and/or on psychotropic medication.	First-time cancer, excluding non-melanoma skin cancer (ICD-10 codes)	ROUTE TO DIAGNOSIS Assesses the association between preexisting psychiatric disorders and routes to diagnosis: death certificate only (DCO), primary care, secondary care, unplanned admission (acute inpatient hospital admission), planned admission, outpatient visit or unknown route.	Without psychiatric conditions.	Population with a psychiatric disorder had an 8.0% lower probability of being diagnosed in primary care and a 7.6% higher probability of being diagnosed through unplanned admissions than those without. Diagnosis initiated in primary care was 37.7% (95% CI 37.1–38.3) for patients with psychiatric disorders and 45.7% (45.3–45.9) for patients without psychiatric disorders. The lowest probability of being diagnosed in primary care was for patients with schizophrenia (41.9, 95% CI: 38.8–45.1) and patients with organic disorders (43.6, 95% CI: 41.8–45.4). Diagnosis through unplanned admissions was 21.8% (95% CI 21.3–22.2) for those with psychiatric disorders and 14.2% (14.0–14.4) for those without. Model was adjusted for sex, age, year of diagnosis, comorbidity, education, ethnicity, cohabitation, region of residence and cancer diagnosis analysis.
Iritani 2011	Japan	Retrospective cohort	Psychiatric hospital	Total sample = 134 cancer patients. With dementia = 50. Without dementia = 84.	• **Cognitive disorders** (dementia) Per-diagnosis analysis • DSM-IV-TR	Cancer	ROUTE TO DIAGNOSIS Reports routes to diagnosis of cancer in people with and without dementia.	Without dementia	Patients with dementia were found to have cancer either accidentally (48%) or by way of another medical evaluation (44%), whereas most patients without dementia (63%) voluntarily sought medical evaluation for cancer (p < 0.001). (No info on adjusted/crude models or multivariate analysis)
**Cardiovascular disease**									
Löppönen 2004	Finland	Cross-sectional, population survey	All inhabitants aged 64 and more in Lieto, Finland Participation rate was 82%.	Total sample = 1252 (with dementia = 112; without dementia = 1140)	• **Cognitive disorders** (dementia). Per-diagnosis analysis Dementia diagnosed by clinical exam according to DSM-IV criteria.	CHD, stroke, hypertension, atrial fibrillation, hypercholesterolaemia, diabetes, hypothyroidism, vit B12 deficiency, anaemia, urinary trait infections.	UNDERDIAGNOSIS Examined the risk of having undiagnosed diseases in older people with and without dementia. Undiagnosed disease was identified through interview, tests and physical examinations.	Without dementia	Patients with dementia were significantly more likely to have undiagnosed hypercholesterolaemia (adj OR = 2.84; 95% 1.22–6.61) and undiagnosed hypothyroidism (OR = 8.16; 95% CI 1.56–42.54) than patients without dementia. Model was adjusted by age and sex.
Castillo-Sanchez 2018	Spain	Cross-sectional	Primary care. Subsample of existing records from the SIDIAP (Information system for research in primary care)	Total = 64,480. With schizophrenia group = 3521. Without schizophrenia but under antipsychotic treatment = 2626. Control group = 58,323.	• **Psychotic disorders** (schizophrenia or using antipsychotic drugs) Per-cluster analysis ICD-10 codes	Hypertension. ICD-10 codes: I10 (essential hypertension), (hypertensive heart disease) I11, I12 (hypertensive renal disease), I13 (hypertensive heart and renal disease), and I15 (secondary hypertension).	UNDERDIAGNOSIS Assess whether there are differences in the proportion of (previously screened) patients who are undiagnosed with hypertension, between patients with schizophrenia, patients without schizophrenia but under antipsychotic treatment, and a control group.	*Without schizophrenia and not on antipsychotic medication.*	*NEGATIVE ASSOCIATION. The schizophrenia group had a lower risk of underdiagnosis of arterial hypertension than the control group (adj OR 0.91; 95% CI: 0.83–0.99; p < 0.05). Authors conclude that a screening programme provides adequate monitoring of this patient group. Model was adjusted by sex, age, and frequentation of visits.*
Lindenfeld 2024	US	Cross-sectional	Electronic health record data from 58 primary care clinics at a large, urban, healthcare system in New York. Patients 18+ years.	7991 had a diagnosis of substance misuse; 307,944 without substance misuse	• **Substance misuse** Per-cluster analysis ICD-10 codes	Hypertension or diabetes (ICD-10 codes for diagnosis.	UNDERDIAGNOSIS Compares rates of diagnosed and undiagnosed hypertension or diabetes among patients with and without a substance misuse.	Without substance use disorder diagnosis	Patients with substance misuse had significantly higher odds of having undiagnosed hypertension adj OR: 1.81; CI: 1.48–2.20) and undiagnosed diabetes (adj OR: 1.93; CI: 1.72–2.16), compared to those without substance misuse (adjusted, multivariate model—adjusted for demographic characteristics and clinical variables). This association was also significant in unadjusted models).
**Other physical health conditions (multiple sclerosis, HIV, encephalopathy)**									
Marrie 2009	US	Retrospective cohort	Self-report registry for patients with multiple sclerosis	Total sample (patients within 2 years of MS diagnosis) = 2375. Subset with mental health comorbidities = 668 (29.4%).	• **Mood disorders** (depression, anxiety disorders) • **Psychotic disorders** (bipolar, schizophrenia) Grouped diagnosis Self-reported.	Multiple sclerosis (self-reported to database but likely to have been diagnosed by a doctor).	LATE-STAGE DIAGNOSIS Compares time from symptom onset to diagnosis, and degree of disability at the point of diagnosis, in those with and without mental health conditions.	Without mental health conditions	Mean difference (SD) delay in diagnosis (years), from age of symptom onset to diagnosis, in people with mental health conditions vs those without: </ = 25 y: 6.3 (4.9–7.6), p < 0.0001; >/ = 25 y–</ = 40 y: 2.0 (1.6–2.5), p < 0.001; >/ = 40 y p = 0.03. The presence of mental health conditions was associated with increased degree of disability at point of multiple sclerosis diagnosis (severe vs mild, adj OR 1.53 (1.16–2.02), CI 95%)
**Autopsy studies: Likelihood of not being diagnosed with physical illness before death**									
Heiberg 2019	Norway	Retrospective cohort	Primary Care/Specialised Care/Death Registry	Total sample (cardiovascular deaths from 2011 to 2016) = 72,451. Subgroup with schizophrenia = 814. Subgroup with bipolar = 673	• **Psychotic disorders** (schizophrenia, bipolar disorder) Per-cluster analysis. ICD codes.	Cardiovascular disease	UNDERDIAGNOSIS Examined the likelihood of being diagnosed with cardiovascular disease prior to cardiovascular death, in people with and without schizophrenia and bipolar.	Individuals without schizophrenia or bipolar	Odds of **not** being diagnosed with cardiovascular disease prior to cardiovascular death: Individuals with schizophrenia were 66% more likely (adj OR: 1.66; 95% CI: 1.39–1.98) than those without; women with bipolar were 38% more likely (adj OR: 1.38; 95% CI: 1.04–1.82); and men with bipolar were equally likely (adj OR: 0.88, 95% CI: 0.63–1.24). Model was adjustment for age at death and comorbidities.
Baillargeon 2011	US	Retrospective cohort.	SEER cancer registry and linked Medicare database.	Total (patients with colon cancer) = 80,670. Subgroup of patients with psychiatric conditions = 20,699.	• **Mood disorders** • **Psychotic disorders** • **Cognitive disorders** (dementia) • **Substance misuse** • Other not fitting into these categories Per-cluster analysis ICD-9-CM codes	Colon cancer (SEER registries)	UNDERDIAGNOSIS Examined association between prior psychiatric diagnosis and diagnosis at autopsy for colon cancer.	Without psychiatric conditions	Participants with a psychiatric diagnosis were more likely to have had colon cancer diagnosed at autopsy (4.4%) than those without (1.1%), p < 0.001. This finding persisted across each of the mental condition subgroups and was particularly pronounced for those with a pre-existing diagnosis of psychosis (7.5%) and those with dementia (8.1%). Unclear if these data were adjusted.
Gupta 2004	US	Retrospective cohort	SEER-Medicare data set (NCI-sponsored individual-level linkage of the clinical data collected by the SEER registries with Medicare billing claims collected for administrative purposes).	Total (patients with colon cancer) = 17,507. Subgroup with dementia = 1184 (6.8%).	• **Cognitive disorders** (dementia) ICD-9 codes	Colon cancer (newly diagnosed)	UNDERDIAGNOSIS Reports colon cancer after death as first record of colon cancer.	Without dementia	Dementia patients were twice as likely to have colon cancer reported after death (i.e., autopsy or death certificate) (adj OR 2.31, 95% CI 1.79–3.00) than patients without dementia. Model adjusted for age, race, marital status, neighbourhood poverty, urban residence, and nondementia medical comorbidity
Puntervold 2021	Denmark	Case-control Autopsy study	SURVIVE study: national autopsy- based cohort study of deceased individuals with suspected mental illness; and Danish National Patient Registry (NPR)	Patients with schizophrenia = 106. No mental health condition = 105	• **Psychotic disorders** (schizophrenia) Per-diagnosis analysis ICD-8 or ICD-10 codes	Somatic comorbidities (most prevalent were chronic pulmonary disease; mild liver disease; cancer, congestive heart failure).	UNDERDIAGNOSIS Identify and compare the somatic comorbidities antemortem and postmortem in autopsied decedents with schizophrenia and with no mental health condition, using the Charlson Comorbidity Index (CCI).	Without mental health condition	*NO SIGNIFICANT ASSOCIATION. The autopsies revealed undiagnosed diseases in both decedents with schizophrenia and no mental health condition. A diagnosis of schizophrenia was correlated with the Charlson Comorbidity Index score antemortem, but not postmortem (antemortem, adj OR 1.880 [1.207–2.928],* p < *0.005; postmortem, adj OR 1.170 [0.828–1.654],* p < *0.374). This suggests that underdiagnosis discovered at the point of death was not statistically significant more in the schizophrenia group.*
**Population-level studies examining the difference in recorded physical health diagnosis between people with and without mental health conditions**									
Olson 2021	Canada (British Columbia)	Matched cohort	Population-based provincial databases	Total sample = 165,289	• **Mood disorders** (Depression, anxiety disorders) • **Psychotic disorders** (schizophrenia, bipolar, multiple personality disorder [now known as dissociative identity disorder]). Per-cluster analysis ICD-9 and ICD-10	Tobacco-related cancer (oropharyngeal, laryngeal, oesophageal, lung and bronchial, acute myeloid leukaemia, stomach, liver, pancreatic, kidney and renal pelvis, cervix, urinary bladder and colorectal).	UNDERDIAGNOSIS Assess risk of being diagnosed with tobacco-related cancer diagnosis in people with and without a mental health condition.	Without mental health conditions (individuals with appendicitis were used as a primary population-proxy control group.)	People with some mental health conditions had a statistically significant lower risk of having a tobacco-related cancer diagnosis compared to people in the comparison group (risk remained when death was treated as competing risk). The authors interpret this finding as indicating systematic under-diagnosis. Depression (HR = 0.81; p < 0.01; 95% CI: 0.73–0.91); anxiety disorders (HR = 0.84; p = 0.02; 95% CI: 0.73–0.97); multiple personality disorder (now known as dissociative identity disorder) (HR = 0.74; p < 0.01; 95% CI: 0.66–0.83). No evidence of a statistically significant difference was found for people with schizophrenia (HR = 0.86; p = 0.40; 95% CI: 0.62–1.21) and bipolar disorder (HR = 0.58; p = 0.12; 95% CI: 0.29–1.14). Matched cohorts on age at diagnosis, sex, year of hospital admission, postal code (as a proxy for socioeconomic status).
Smith 2013	Scotland (UK)	Cross-sectional	312 primary care practices in Scotland	People with schizophrenia = 9677. Controls = 1,414,701.	• **Psychotic disorders** (schizophrenia spectrum) Per-cluster analysis Read codes	Physical health comorbidity	UNDERDIAGNOSIS Assess nature of physical health comorbidities in people with schizophrenia and related psychoses compared with controls.	Without schizophrenia and not on antipsychotic medication.	People with schizophrenia had lower recorded rates of cardiovascular disease, including atrial fibrillation (OR 0.62, 95% CI 0.51–0.73), hypertension (OR 0.71, 95% CI 0.67–0.76), coronary heart disease (OR 0.75, 95% CI 0.61–0.71) and peripheral vascular disease (OR 0.83, 95% CI 0.71–0.97). ORs were adjusted for age, sex, and deprivation score. Authors interpret this as a systematic under-recognition and undertreatment of cardiovascular disease in people with schizophrenia.
Crump 2013	Sweden	Retrospective cohort	Any primary or secondary diagnosis in the Swedish Outpatient Registry, Swedish Hospital Registry, Swedish Pharmacy Registry	Patients with Schizophrenia = 82,773,490 (women) + 4787 (men)	• **Psychotic disorders** (schizophrenia, being on antipsychotic medications) Per-cluster analysis ICD-10 codes.	Hypertension (I10), ischemic heart disease (I20–I25), stroke (I60–I66), cancer (C00–C97), diabetes mellitus (E10–E14), lipid disorders (E78), influenza or pneumonia (J09–J18), chronic obstructive pulmonary disease (COPD) (J41–J44), and liver disease (K70–K77).	UNDERDIAGNOSIS Examined HR for the association between schizophrenia and selected health outcomes, compared to people without schizophrenia.	Without schizophrenia	Among all people who died from ischemic heart disease or cancer, schizophrenia patients were less likely than others to have been diagnosed previously with these conditions (for ischemic heart disease, 26.3% compared with 43.7% (p < 0.001); for cancer, 73.9% compared with 82.3% (p < 0.005). After restricting the analysis to people who were previously diagnosed, schizophrenia was only modestly associated with ischemic heart disease mortality (adj HR = 1.36, 95% CI = 1.05–1.77) and was no longer associated with cancer mortality (adj HR = 1.04, 95% CI = 0.87–1.24). Authors interpret this as underdiagnosis.
Avgerinou 2024	UK	Retrospective cohort (population-based)	UK routine primary care data (IQVIA Medical Research Database (IMRD)). Study population 50+ years.	50,006 with psychotic disorders; 397,474 without psychotic disorders (age- and sex- matched)	• **Psychotic disorders** (schizophrenia, bipolar or other psychosis) Per-cluster diagnosis Read codes	Osteoporosis (OP) and fragility fracture (FF)	UNDERDIAGNOSIS Compares the incidence of recorded OP diagnosis and FF between people aged ≥50 years with SMI and those without. Uses the incidence data to examine the FF: OP ratio—ratio of diagnosed fragility fractures to diagnosed osteoporosis as a potential indicator of underdiagnosis of osteoporosis.	Without psychotic disorder	Amongst men with psychotic disorders there were more than twice as many with a FF diagnosis than with OP diagnosis FF: OP = 2.10. For men without psychotic disorders FF: OP = 1.89. For women with SMI, the FF: OP ratio was 1.56, whereas for women without SMI the ratio was 1.11. The authors conclude that these figures suggest that osteoporosis is underdiagnosed both in men and women with SMI (with a relatively more pronounced effect in women with SMI compared to non-SMI). (Significance data reported for the FF: OP ratio data. Only for the recorded incidences of FF and OP. Incidence models fully adjusted for age, sex, social deprivation, smoking, alcohol, and Body Mass Index).

One of the included articles (O'Rourke et al.^30^) separately reported findings relating to both diagnostic error and diagnostic disparities, and is therefore included in both groups.

### Narrative synthesis

#### Studies suggestive of diagnostic error

Fifteen studies assessed the risk of diagnostic problems that occur after patients' presentation to services, and thus suggest diagnostic error. Of these, fourteen found some evidence that having a mental health condition is associated with greater risk of diagnostic error, and one study found no evidence of association. Only one study,^31^ however, utilised information (malpractice claim data) confirming that a diagnostic error had occurred through independent, definitive subsequent review, in line with the definition of provided by Newman-Toker et al.^17^ The other 13 studies presented findings strongly suggestive of diagnostic error (as reflected in the authors’ conclusions), but without confirmation through “a subsequent definitive test or finding”.^17^ Table 1 reports how diagnostic errors were operationalised in each study.

Twelve studies were retrospective cohort,^30^^,^^32^, ^33^, ^34^, ^35^, ^36^, ^37^, ^38^, ^39^, ^40^, ^41^, ^42^ two were experimental,^43^^,^^44^ and one was a case–control.^31^ For studies quality-appraised with the Newcastle–Ottawa scale, the RoB was low (n = 8)^30^, ^31^, ^32^^,^^34^, ^35^, ^36^, ^37^, ^38^^,^^41^^,^^42^ or moderate (n = 5)^30^^,^^33^^,^^35^^,^^39^^,^^40^; for the two studies assessed with RoB 2, ‘some’ risk of bias was identified^43^^,^^44^ (Appendix 3). All but one study^40^ reported adjusted models.

Eight studies were from the US,^30^^,^^34^^,^^36^, ^37^, ^38^^,^^40^^,^^43^^,^^44^ three from the UK,^32^^,^^33^^,^^41^ and one each from Sweden,^31^ Switzerland,^39^ the Netherlands,^42^ and Denmark.^35^ Most studies included older age groups and were mixed by sex and ethnicity.

Seven studies focused on cancer^30^^,^^32^, ^33^, ^34^, ^35^^,^^41^^,^^42^ (colorectal, lung, oesophageal, and breast cancer), four on cardiovascular problems^36^, ^37^, ^38^^,^^44^ (hypertension, stroke, myocardial infarction, aortic dissection, and others), and four on other individual (multiple sclerosis,^39^ sepsis,^40^ and pernicious anaemia^43^) or grouped^31^ physical health conditions.

Eight studies focused on delayed diagnosis,^30^^,^^32^, ^33^, ^34^, ^35^^,^^39^^,^^41^^,^^42^ four on missed diagnosis,^36^^,^^37^^,^^40^^,^^44^ one on both,^38^ one on missed diagnosis or misdiagnosis,^43^ and one on unspecified diagnostic errors.^31^ Delayed diagnosis was uniformly operationalised as the time interval from the recording of concerning symptoms (at presentation) to recording of a diagnosis. Missed diagnosis was operationalised in more heterogeneous ways (Table 1), including identification of concerning symptoms (in prior visits) followed by discharge without investigation^37^; re-admission to hospital following a treat-and-release emergency department encounter associated with a benign diagnosis^40^; and absence of a diagnosis after a defined period from the recording of concerning symptoms.^38^

##### Cancer

Seven studies (all delayed diagnosis studies) focused on of cancer. Four focused on colorectal cancer:

• Benitez Majano et al.^32^ found longer diagnostic intervals (from consultation to diagnosis) for people with mental health conditions than without: 466 days (95% CI: 413–519) vs 365 days (95% CI: 289–442) at the 75th centile (p < 0.001); and 224 days (95% CI: 159–290) vs 126 days (95% CI: 94–158) at the 50th centile (p = 0.003) (adjusted model).
• Mounce et al.^41^ found that co-occurring anxiety or depression was associated with longer diagnostic intervals: a nine-day delay (95% CI: 3–17, p = 0.007), compared to those without anxiety or depression (adjusted model).
• Van Hout et al.^42^ found that psychiatric comorbidities were associated with delay, by the GP, in referring patients to specialist care: adjusted OR 3.97 (95% CI: 1.14–13.85). No statistically significant association was found in the period from referral to specialist to histological diagnosis.
• Walter et al.^33^ found that comorbid anxiety or depression was associated with a longer interval between first presentation and diagnosis. Those with mental health conditions were diagnosed 0.80 (95% CI: 0.71–0.90) times as quickly as those without (p < 0.001; adjusted HR).

The remaining studies focused on breast, oesophageal, and lung cancer. Iglay et al.^34^ found that patients with comorbid anxiety/depression had an 11% increased risk of breast cancer diagnostic delay of at least 90 days from symptom recognition (adjusted RR: 1.11; 95% CI: 1.00–1.23). However, when considering those with *any* mental health condition (undifferentiated by diagnosis of mood or psychotic disorders), no statistically significant difference was found in the risk of diagnostic delay at 60 and 90 days. O'Rourke et al.^30^ found that having a psychiatric illness, and a specific diagnosis of depression, were both predictive of delayed diagnosis of oesophageal cancer (adjusted HR: 0.605 (0.424–0.862) and 0.622 (0.425–0.910) respectively). Patients with psychiatric conditions experienced a median diagnostic delay of 90 days (IQR 20–162) vs 35 days (IQR 0–76) for those without (p = 0.001). Finally, Iachina et al.^35^ found no significant difference in duration of diagnostic process for non-small cell lung cancer between those with and without depression (adjusted HR: 0.99; 95% CI: 0.90–1.09).

##### Cardiovascular illness

Sharp et al.^36^ found higher likelihood of missed diagnosis of myocardial infarction in the emergency department in those with mental health conditions (mood-related and schizophrenia-related) (adjusted OR: 1.48, 95% CI: 1.23–1.77). This difference was not significant in those with substance misuse. Waxman et al.^37^ found that depression and dementia were independently associated with risk of five cardiovascular conditions being missed: adjusted OR (95% CI) estimates for depression were >1 for all five conditions, and for dementia for four of five conditions. Finally, Byrd et al.^38^ found that the probability of receiving a diagnosis of hypertension was *not* significantly different in patients with anxiety and depression compared to patients without these diagnoses (adjusted HR for anxiety and depression 0.94, 95% CI: 0.89–1.00), but it was lower for those with anxiety alone (adjusted HR: 0.93, 95% CI: 0.88–0.99) and depression alone (adjusted HR: 0.93, 95% CI: 0.90–0.97) compared with patients with neither condition. Moreover, median days to diagnosis were greater in patients with depression and anxiety than in patients without (31 days, IQR: 0–174 vs 5 days, IQR: 0–126, p < 0.001).

##### Other physical health conditions

Barin et al.^39^ examined delayed diagnosis of multiple sclerosis. Modelling time from first contact with healthcare to first specialist evaluation, and from specialist evaluation to diagnosis, they found depression (as concomitant first symptom) was associated with prolonged time from specialist evaluation to diagnosis (adjusted OR: 0.46, 95% CI: 0.24–0.91). Nassery et al.^40^ examined antecedents of sepsis misdiagnosis in the emergency department.^45^ Comparing observed and expected (O:E) rates of sepsis, they found that altered mental status was one of the two strongest predictors of downstream sepsis hospitalisation after a treat-and-release episode (O:E 2.86, 95% CI: 2.04–4.00), alongside fluid and electrolyte disorders. Fernholm et al.’s^31^ registry-based study examined factors associated with risk of preventable harms (of which 46% involved diagnostic error of somatic disease), and found that patients with psychiatric illness had a nearly two-fold risk (adjusted OR: 1.96, 95% CI: 1.76–2.19, p < 0.001). Of all reported cases of preventable harm, 46% involved diagnostic error of somatic disease.

##### Experimental, vignette-based studies

Two studies were methodologically distinctive. Rather than analysing patient data, they utilised experimental designs to explore whether the presence of co-occurring mental health conditions affected clinicians’ diagnostic accuracy.

Isbell et al.^43^ presented physicians with a vignette of a patient with pernicious anaemia, and randomised them to diagnose a patient with or without comorbid depression. Diagnostic accuracy (timely and correct diagnosis) was lower among physicians exposed to the depression vignette, though this was not statistically significant (59.4% vs 40.7%; p = 0.15). Accuracy was significantly lower in the depression condition only when physicians ordered fewer tests (1SD below mean; OR: 0.103, p = 0.028).

McDonald et al.^44^ exposed nurses to a vignette describing a patient with possible myocardial infarction (MI), randomised across three groups (co-occurring psychosis, co-occurring anxiety, no mental health conditions). Nurses in the psychosis group were less likely to predict MI than those in the control group, suggesting missed diagnosis: mean probability of patient being diagnosed with MI was 35% (SD: 18.2) in the psychosis group vs 50.6% (SD: 28.2) in the control group. Nurses in the anxiety group predicted MI slightly less than the control group but more than the psychosis group (mean probability 49.5%, SD: 19.29).

##### Analysis by clusters of mental health conditions

**Fig. 3:**
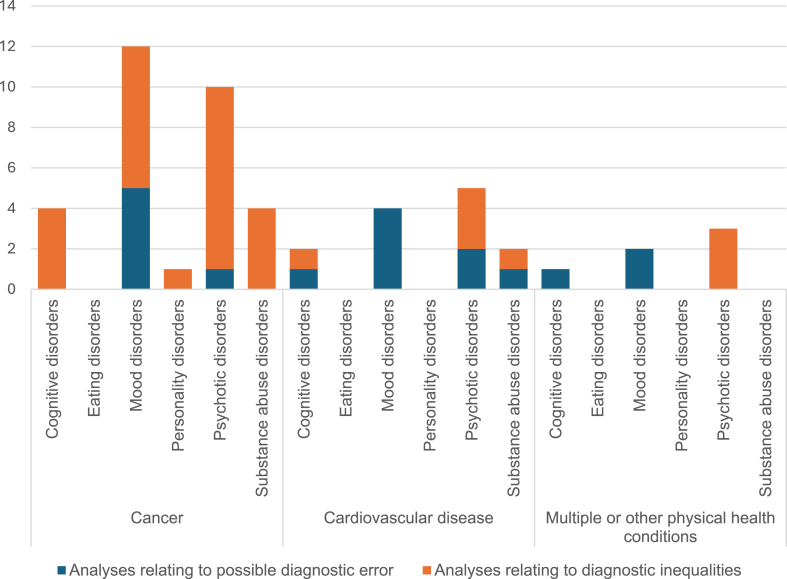
Discrete analyses across all papers with a robust mental health comparator group, by mental health cluster, physical health condition and type of study.

To provide a meaningful synthesis, we organised mental health conditions examined by the studies into six clusters (aligned with ICD-11 classifications)^46^: mood disorders (depression and anxiety disorders); psychotic disorders (schizophrenia, schizoaffective disorder, bipolar, and other psychoses); cognitive disorders (e.g. dementia); personality disorders; eating disorders; and substance misuse. Studies varied in whether they reported estimates for psychiatric conditions as a unified group (‘grouped analysis’), or performed separate analyses for clusters of mental health conditions, such as mood disorders (‘per-cluster analysis’) or individual mental health diagnoses (‘per-diagnosis analysis’) (Table 1). Fig. 3 shows the total number of discrete analyses performed across all papers included in the narrative review.

Three studies^30^, ^31^, ^32^ conducted grouped analyses only. Seven studies^33^^,^^34^^,^^36^^,^^38^^,^^41^^,^^42^^,^^44^ performed per-cluster analysis. Of these:

• Four^33^^,^^38^^,^^41^^,^^42^ examined the relationship between mood disorders (depression and/or anxiety) and risk of diagnostic error, identifying a significant positive association;
• Two^34^^,^^44^ compared mood disorders and psychotic disorders: Iglay et al. found that only comorbid anxiety and depression (not psychotic disorders) were significantly associated with increased risk of diagnostic delay of breast cancer,^34^ whilst McDonald et al. found that psychotic disorders (not anxiety) were significantly associated with the risk of missed diagnosis of MI.^44^
• One^36^ found slightly higher proportions of missed MI in those with anxiety and other mood disorders than with psychotic conditions (schizophrenia-related) and substance misuse.

Five studies^35^, ^36^, ^37^^,^^39^^,^^40^^,^^43^ conducted per-diagnosis analyses. Four^35^^,^^37^^,^^39^^,^^43^ included a specific analysis for depression, of which three^37^^,^^39^^,^^43^ found an association between with increased risk of diagnostic error (albeit one was non-significant); one^35^ found no statistically significant association. Overall, depression was the most frequently explored mental health condition (in 12/15 studies), followed by anxiety (nine studies) and schizophrenia (four studies).

#### Wider diagnostic inequalities

Twenty-three studies^30^^,^^47^, ^48^, ^49^, ^50^, ^51^, ^52^, ^53^, ^54^, ^55^, ^56^, ^57^, ^58^, ^59^, ^60^, ^61^, ^62^, ^63^, ^64^, ^65^, ^66^, ^67^, ^68^ examined the risk of diagnostic inequalities experienced by people with mental health conditions. These disparities are likely to be the result of multiple factors, including patient-related factors (such as late presentation) and professional- or system-related factors. The design of these studies did not allow us to isolate post-presentation factors from wider influences.

Sixteen studies^30^^,^^47^^,^^50^, ^51^, ^52^, ^53^^,^^56^^,^^58^, ^59^, ^60^, ^61^^,^^63^, ^64^, ^65^^,^^67^^,^^68^ found some evidence that having a mental health condition is significantly associated with greater risk of diagnostic inequalities; two^49^^,^^66^ found an association but did not report significance data. Four^48^^,^^54^^,^^55^^,^^62^ found no statistically significant association; one^57^ found a negative association.

Seventeen were cohort studies (16 retrospective,^30^^,^^47^, ^48^, ^49^, ^50^^,^^52^, ^53^, ^54^, ^55^^,^^58^, ^59^, ^60^, ^61^^,^^65^^,^^66^^,^^68^ one matched cohort^63^), four were cross-sectional,^56^^,^^57^^,^^64^^,^^67^ and two were case–control studies.^51^^,^^62^ Nine studies were from the US,^30^^,^^47^, ^48^, ^49^^,^^55^^,^^58^^,^^60^^,^^61^^,^^67^ three from the UK,^54^^,^^64^^,^^66^ two from Spain,^51^^,^^57^ two from Denmark,^53^^,^^62^ two from New Zealand,^50^^,^^68^ and one each from Japan,^52^ Finland,^56^ Norway,^59^ Sweden,^65^ and Canada.^63^ RoB was low for 15 studies,^47^^,^^48^^,^^50^^,^^51^^,^^53^, ^54^, ^55^^,^^57^^,^^59^, ^60^, ^61^, ^62^^,^^65^^,^^67^^,^^68^ moderate for seven studies,^30^^,^^49^^,^^52^^,^^56^^,^^63^^,^^64^^,^^66^ and high for one study^58^ (Appendix 4).

Eleven studies^30^^,^^47^, ^48^, ^49^, ^50^, ^51^, ^52^, ^53^, ^54^, ^55^^,^^68^ examined cancer-related diagnostic inequalities. Four^30^^,^^47^^,^^50^^,^^51^ found evidence that people with mental health conditions were more likely to have cancer diagnosed at a later stage, and one^49^ found an association but did not report significance data. Three studies^52^^,^^53^^,^^68^ found evidence that having a mental health condition was associated with higher likelihood of being diagnosed with cancer via an unplanned or emergency diagnostic route (route-to-diagnosis studies, Box 1). Three studies found no statistically significant association between cancer diagnostic inequalities and mental health condition.^48^^,^^54^^,^^55^ Three studies assessed risk of underdiagnosis of cardiovascular disease. Löppönen et al.^56^ and Lindefeld et al.^67^ found that this risk was associated with having a mental health condition, while Castillo-Sanchez et al.^57^ found the opposite (people with schizophrenia had reduced risk of underdiagnosis of hypertension). One study^58^ identified a positive association between having a mental health condition and the risk of late-stage diagnosis of multiple sclerosis.

The remaining eight articles included two methodologically distinctive sub-sets. Four^59^, ^60^, ^61^, ^62^ reported findings from autopsies. Three^59^, ^60^, ^61^ found some evidence that having a mental health condition was associated with increased risk of undiagnosed physical health problems (cancer and cardiovascular illness) at death, and one^62^ found no statistically significant difference between groups. Four studies^63^, ^64^, ^65^, ^66^ used population-level data to examine differences in diagnostic patterns between people with and without mental health conditions. Where people with mental health conditions were less likely to have a specific diagnosis, the authors inferred that systematic underdiagnosis existed. All four studies found evidence that certain mental health conditions were associated with underdiagnosis of physical health problems (but, in one,^66^ significance data were not reported). Olson et al.^63^ found this association in depression, anxiety, and dissociative identity disorder, but not for schizophrenia and bipolar.

##### Analysis by mental health condition

Most diagnostic inequalities studies examined multiple mental health conditions; four studies performed grouped analyses only, fifteen performed per-cluster analyses and five offered per-diagnosis analyses.

The most widely examined cluster was psychotic disorders (Fig. 3), included in 17 studies^30^^,^^49^, ^50^, ^51^^,^^53^, ^54^, ^55^^,^^57^, ^58^, ^59^, ^60^^,^^62^, ^63^, ^64^, ^65^, ^66^^,^^68^ (14 studies assessed conditions on the schizophrenia spectrum and seven looked at bipolar). Of these studies, twelve^30^^,^^49^, ^50^, ^51^^,^^53^^,^^58^, ^59^, ^60^^,^^64^, ^65^, ^66^^,^^68^ identified a positive association between psychotic disorders and increased likelihood of exposure to diagnostic inequalities, four^54^^,^^55^^,^^62^^,^^63^ found no statistically significant association, and one found a negative association.^57^

Mood disorders were the second-most examined cluster (included in nine studies^30^^,^^48^^,^^51^^,^^53^, ^54^, ^55^^,^^58^^,^^60^^,^^63^): five studies examined depression and six anxiety-related disorders. Of these studies, four^51^^,^^53^^,^^60^^,^^63^ found a positive association between having a mood disorder and increased risk of exposure to diagnostic inequalities and three^48^^,^^54^^,^^55^ did not (two studies^30^^,^^58^ performed grouped analysis only, meaning that the specific association between diagnostic inequalities and the mood disorder cluster remains unknown).

## Discussion

**Fig. 4:**
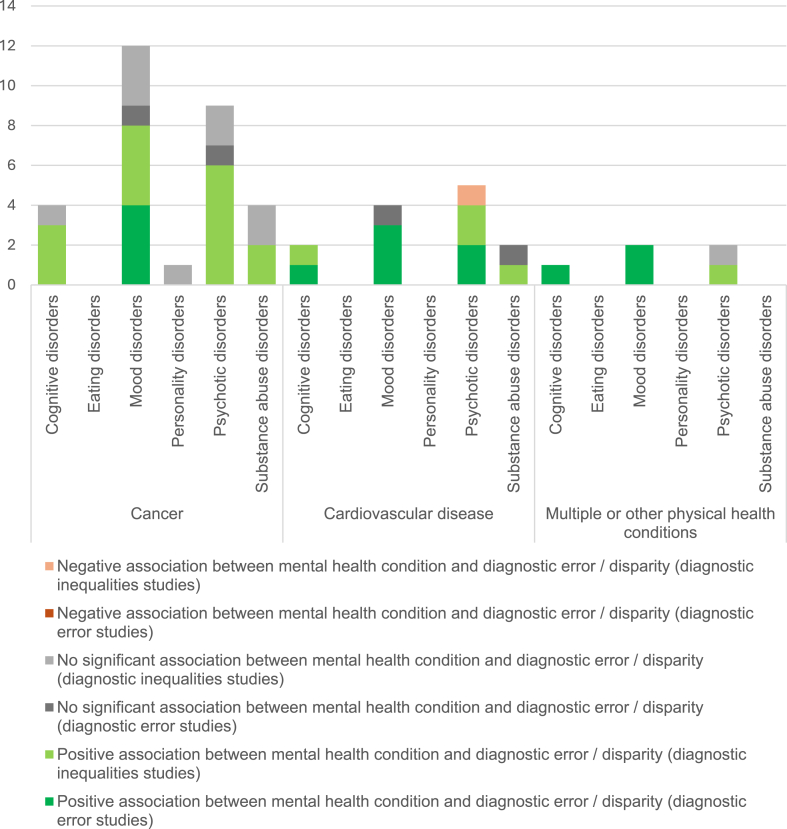
Discrete analyses across all papers with a robust mental health comparator group including statistical significance data by mental health cluster, physical health condition, type of study and direction of association.

A substantial body of research indicates that people with mental health conditions suffer from worse physical health than those without.^2^^,^^4^ Our systematic review contributes to this evidence by assessing the role of diagnostic inequalities. Of 37 included studies with a robust mental health comparator group, 29 found that having one or more mental health conditions is associated with a statistically significant increased risk of having a physical health problem undiagnosed or diagnosed late. Three additional studies made a similar claim but did not report on statistical significance. Overall, this offers convincing evidence that diagnostic inequalities affect this population (Fig. 4 illustrates the discrete analyses by mental health cluster conducted across all included studies).

The most striking and easiest-to-interpret evidence comes from the 15 studies suggestive of diagnostic error (Table 1). Thanks to designs that included measures of diagnostic process as well as diagnostic endpoints, these studies could exclude patients’ late or non-presentation to services as a possible cause of inequalities. Fourteen of these studies confirmed that individuals with mental health conditions are more likely to be exposed to diagnostic errors for their physical health—demonstrating the contributing role of professional- and service-related factors in producing diagnostic inequalities. This important finding is confirmed by the broader health inequalities literature, which consistently identifies individuals with mental health conditions as at risk of lower-quality care,^11^^,^^69^ stigma,^27^ and diagnostic overshadowing.^21^

A larger number of studies (n = 23) pointed to increased diagnostic inequalities for people with mental health conditions (identified by sixteen studies), but did not allow inference on the mechanisms underpinning these inequalities (for various reasons, including diagnostic data analysis that was a subset of a broader analysis plan, and was therefore more limited in scope, and use of population-level datasets that did not contain granular information about patients’ diagnostic journeys). However, the authors of these studies did present speculative hypotheses on the reasons for the inequalities they identified, usually pointing to a combination of epidemiological, patient-related, and healthcare-related factors. For example, Olson et al.^63^—who identified evidence indicative of underdiagnosis of tobacco-related cancers—reasoned that the cancers they examined are usually highly symptomatic and are therefore unlikely to be ignored by patients. They highlighted that stigma towards psychiatric patients is likely to be a mechanism underpinning underdiagnosis, and identified lower participation in screening and primary care appointments as additional possible contributors.

Five studies (one diagnostic error study and four diagnostic inequalities studies) found no difference in diagnostic inequalities between patients with and without mental health conditions.^35^^,^^48^^,^^54^^,^^55^^,^^62^ A suggested explanation was that more frequent access to healthcare, along with additional surveillance measures and behavioural interventions for this group, may offset any negative effects of psychiatric conditions on diagnosis.^48^^,^^55^ Only one study found a negative association between having a mental health condition and the risk of diagnostic inequalities, finding that patients with schizophrenia had lower risk of underdiagnosis of arterial hypertension than the comparator group.^57^ The authors attributed this to the fact that patient in their sample had benefitted from screening and preventive management, which appeared to be efficacious.

Forty-two studies, reported in Appendix 2, did not have a robust mental health comparator group and were excluded from the narrative synthesis. Most of these measured diagnostic disparities through reappraisal: they compared the rates of already-known diagnoses with the rates of new diagnoses identified at the point of further testing. This approach has several limitations: it does not consider the possible late onset of the health problem or the circumstances of the previous evaluation, and it suffers from ‘hindsight bias’.^70^ Moreover, without a comparator group or a valid estimate of the expected underdiagnosis rate in the general population, the findings are difficult to interpret.

The diagnostic inequalities identified by this review have potentially serious clinical consequences. Late-stage diagnosis of cancer may lead to delays in commencing treatment that could substantially impact outcomes. Similarly, underdiagnosis of cardiovascular conditions has implications for timely commencement of active treatment or secondary prevention. Tailored improvement actions must therefore be considered.

The diagnostic error studies clearly identify the contribution of professional- and service-related factors contribute to delayed or missed diagnosis. Improvement efforts that place the onus of behavioural change solely on patients cannot address these issues. A key focus for practice should therefore be developing meaningful improvement interventions targeted at clinicians and healthcare providers. Tackling diagnostic overshadowing, stigma, and unconscious bias in healthcare professionals is vital. Improvement efforts should also address organisational silos and ways of working that make it harder to meet the complex needs of patients with comorbid physical and mental health conditions.

Research on health inequalities in people with mental health conditions has traditionally focused on patients with SMIs, reflecting this population's marked excess mortality. In England, primary care screening measures are in place to promote the timely identification of common and manageable conditions like hypertension in people with SMIs.^71^ Our review provides evidence that patients with more common mental health conditions (such as depression and anxiety) are also exposed to diagnostic inequalities, including diagnostic error. Consideration might be given to the benefits and risks of extending screening programmes to include other mental health presentations.

Diagnostic inequalities are notoriously complex to measure and address.^14^ As recently highlighted,^70^ advancing this field requires understanding of the factors contributing to suboptimal diagnosis. However, most included studies did not explicitly consider the stage of the diagnostic process at which disparities occurred, and future research might address this more carefully.

Our review only included studies that explicitly reported measures of diagnostic error or inequality. We identified several studies reporting on under-presentation to healthcare or screening services, but these seldom included, or were linked to, data on diagnostic patterns. Future studies should connect these two bodies of research, clarifying the impact of different factors on diagnostic disparities to inform intervention.

Across our dataset, we found that diagnostic constructs were often poorly defined, and definitions of diagnostic inequalities were inconsistent. We recommend further standardisation in reporting diagnostic inequalities, following the principles highlighted by Giardina et al.^15^^,^^18^ and the National Academies of Science, Engineering and Medicine^13^ to support future synthesis efforts.

In terms of clinical focus, most studies with a robust comparator group examined delayed, late-stage, or unplanned diagnosis of cancer (18 of 37 studies). Timely cancer diagnosis is indeed an important public health concern. However, the mortality gap in patients with SMIs appears to be associated particularly with cardiovascular problems, with recent studies estimating the risk of cardiovascular mortality in patients with SMI being up to five times higher than the general population.^72^ Yet only six of our 37 studies related to diagnostic inequalities for cardiovascular illness.

With regard to mental health conditions, the diagnostic error studies (Table 1) focused largely on depression and anxiety: psychotic disorders were somewhat under-represented. Since psychotic disorders include some of the most stigmatised mental health conditions (such as schizophrenia^73^) with pronounced excess mortality, further studies should target these diagnoses. Studies relating to eating disorders and personality disorders were underrepresented across diagnostic errors and diagnostic inequalities studies, while studies relating to substance abuse were found predominantly in the diagnostic inequalities papers.

While the results offer clear evidence of a disadvantage, they should be interpreted with some caution. We included studies that examined diagnostic inequalities using a range of definitions, methods, and study designs; although this enabled comprehensive assessment of the evidence, it also meant that no pooled analysis was possible and that weighing studies by quality would not add meaningful insights. Most of the included studies are observational. A minority of studies did not report adjusted models, and so did not account for the impact of confounders. Studies in languages other than English were not included, and our search was limited to the four most relevant bibliographic databases.

There are also limitations in our operationalisation of diagnostic error: only one study^31^ utilised claim data that enabled confirmation that a diagnostic error had definitely occurred.^17^ The remaining studies utilised data that were strongly suggestive of diagnostic error (as reflected in the authors’ conclusions) but lacked “a subsequent definitive test or finding”.^17^ However, use of administrative data, including claims data, for research purposes is itself subject to limitations,^74^ including cohorts that may not be representative of wider populations, missing data, and misclassification. For mental health conditions administrative data may be especially limited in validity.^34^^,^^60^

We did not include studies examining diagnostic inequalities related to physical health in people with intellectual or learning disability. This is a well-known public health concern^75^ that warrants further systematic assessment. Finally, some conditions such as delirium and dementia sit at the intersection of physical and mental health; while in our analysis they were clustered as mental health conditions (in light of their psychological symptoms), we acknowledge this simplification.

Our study advances the evidence base on the presence of diagnostic disparities for physical health problems among people with mental health conditions, including errors occurring after presentation indicative of causes located within healthcare provision. Further investigation of the precise nature of these causes is vital to inform design of interventions to address them.

## Contributors

EL developed the idea for the study and was responsible for the decision to submit the manuscript. SK led the planning of the review methodology, finalised a protocol (with input from EL, AP, and GM), and completed PROSPERO registration. The search terms were defined by EL, SK, IK, AP, and NR. IK designed and conducted the search strategy, exported the results, and removed the duplicates. Abstract screening was conducted by SK, EL, NR, AO, JG, ES, SW, SC. Data extraction was completed by SK, EL, NR, AO, ES, SW, SC, and JG, and reviewed for accuracy by EL and SK. EL and SK had access to and verified the underlying data. EL and SK produced the first draft of the manuscript and tables respectively, and all authors contributed to subsequent revisions. All authors read and approved the final version of the manuscript and agreed on its submission for publication.

## Data sharing statement

No data was collected for this study. The PROSPERO protocol is available at https://www.crd.york.ac.uk/prospero/display_record.php?RecordID=375892.

## Declaration of interests

Graham Martin is a trustee at the Nuffield Trust.
