# Supplementary material for: Diagnostic inequalities relating to physical healthcare among people with mental health conditions: a systematic review
Source: eClinicalMedicine. 2025 Jan 10;80:103026. doi: 10.1016/j.eclinm.2024.103026 (PMC11773261; doi:10.1016/j.eclinm.2024.103026)
Supplement: Appendix 1 [file mmc1.docx]

**Appendix 1. Search strategy**

Summary of results

|  | No. of hits 21st November 2022 (searching 2002-21^st^ Nov 2022) | Update search, No. of hits searching Sept 2022-Sept 2023 | Update search, No. of hits searching Sept 2023-Sept 2024 | Combined hits screened |
| --- | --- | --- | --- | --- |
| MEDLINE via Ovid | 4985 | 314 | 347 |  |
| Embase via Ovid | 5791 | 800 | 774 |  |
| PsycInfo via Ebsco | 2695 | 104 | 88 |  |
| CINAHL via Ebsco | 2785 | 131 | 152 |  |
| **Total** | **16256** | **1349** | **1361** | **18966** |
| **Deduplicated** | **11317** | **1083** | **1008** | **13408** |

Limits

Publication year: 2002 to 21^st^ Nov 2022. Updated searching from 1^st^ Sept 2022 to 31^st^ Sept 2023. Updated again searching 1^st^ September 2023 to 18^th^ September 2024.

Language: English

Publication types excluded: letter, editorial, comments, books, book chapters, dissertations

Full search terms for each database

# **MEDLINE**

Ovid MEDLINE(R) and Epub Ahead of Print, In-Process, In-Data-Review & Other Non-Indexed Citations, Daily and Versions

1 exp Mental Disorders/ or exp Psychotic Disorders/ 1400266

2 (Mental disorder* or mental* ill* or mental health problem* or psychosis* or (psychiatric adj (disease* or disorder* illness* or diagnosis* or complaint* or symptom*))).ti,ab,kw,kf. 174685

3 (schizophren* or bipolar*).ti,ab,kw,kf. or bipolar disorder/ or schizophrenia/ or (delirum or "altered mental status").ti,ab,kw,kf. 236514

4 (disease pair analysis or symptom-disease pair analysis).ti,ab,kw,kf. 6

5 (anxiety disorder* or mood disorder* or major depression or personality disorder* or affective disorder).ti,ab,kw,kf. 105289

6 exp Anxiety Disorders/ or exp Depression/ or exp Mood Disorders/ or exp Personality Disorders/ 361333

7 1 or 2 or 3 or 4 or 5 or 6 1618043

8 (overshadow* or (diagnos* adj3 (error* or mistake*)) or misdiagnos* or diagnostic blindspot* or diagnostic blind spot* or undiagno* or underdiagnos* or (diagnos* adj3 (late or delay* or dispari* or inaccura* or oversight* or pitfall*)) or ((complete or incomplete) adj3 physical examination) or underrecogni* or undertreat* or under recogni* or undetect* or (diagnos* adj1 gap*) or misinterpret* or mask* or masquerad*).ti,ab,kw,kf. 334681

9 substantial* disadvantag*.ti,ab,kw,kf. 79

10 exp Diagnostic Errors/ or exp Delayed Diagnosis/ 129558

11 (treatment pattern* or treatment gap).ti,ab,kw,kf. 7699

12 8 or 9 or 10 or 11 451765

13 (physical health or physical* ill* or physical disorder* or co-occuring or co-occur* or co-morbid* or comorbid* or multimorbid* or multi-morbid* or concomitant or "secondary to medical illness" or concurrent chronic condition* or multiple chronic condition*).ti,ab,kw,kf. 505941

14 exp Comorbidity/ or exp Multimorbidity/ 125267

15 ((myocardial infarction or heart attack or cardiovascular disease* or cardiovascular disorder* or diabetes or metabolic disease* or metabolic disorder* or infection disease* or communicable disease* or cancer* or neoplasm*).ti,ab,kw,kf. or (exp myocardial infarction/ or exp cardiovascular diseases/ or exp heart diseases/ or exp diabetes mellitus/ or exp metabolic diseases/ or exp communicable disease/ or exp neoplasm/) or exp Sepsis/ or sepsis.ti,ab,kw,kf.) and (diagnos*.ti,ab,kw,kf. or di.fs.) 2227270

16 13 or 14 or 15 2719956

17 7 and 12 and 16 6793

18 (letter or editorial or comment).pt. 2111315

19 17 not 18 6632

20 limit 19 to english language 5961

21 limit 20 to yr="2002 -Current" 4985

# **EMBASE**

1 exp *Mental Disease/ or exp *psychosis/ 1509122

2 (Mental disorder* or mental* ill* or mental health problem* or psychosis* or (psychiatric adj (disease* or disorder* illness* or diagnosis* or complaint* or symptom*))).ti,ab. 214879

3 (schizophren* or bipolar*).ti,ab. or exp *bipolar disorder/ or *schizophrenia/ or (delirum or "altered mental status").ti,ab. 285818

4 (disease pair analysis or symptom-disease pair analysis).ti,ab. 14

5 (anxiety disorder* or mood disorder* or major depression or personality disorder* or affective disorder).ti,ab. 139929

6 exp *Anxiety Disorder/ or exp *Depression/ or exp *Mood Disorder/ or exp *Personality Disorder/ 402749

7 1 or 2 or 3 or 4 or 5 or 6 1698901

8 (overshadow* or (diagnos* adj3 (error* or mistake*)) or misdiagnos* or diagnostic blindspot* or diagnostic blind spot* or undiagno* or underdiagnos* or (diagnos* adj3 (late or delay* or dispari* or inaccura* or oversight* or pitfall*)) or ((complete or incomplete) adj3 physical examination) or underrecogni* or undertreat* or under recogni* or undetect* or (diagnos* adj1 gap*) or misinterpret* or mask* or masquerad*).ti,ab. 461205

9 substantial* disadvantag*.ti,ab. 86

10 exp *Diagnostic Error/ or exp *Delayed Diagnosis/ 16911

11 (treatment pattern* or treatment gap).ti,ab. 16195

12 8 or 9 or 10 or 11 484462

13 (physical health or physical* ill* or physical disorder* or co-occuring or co-occur* or co-morbid* or comorbid* or multimorbid* or multi-morbid* or concomitant or "secondary to medical illness" or concurrent chronic condition* or multiple chronic condition*).ti,ab. 782543

14 *Comorbidity/ or exp *Multimorbidity/ 25494

15 ((myocardial infarction or heart attack or cardiovascular disease* or cardiovascular disorder* or diabetes or metabolic disease* or metabolic disorder* or infection disease* or communicable disease* or cancer* or neoplasm*).ti,ab. or (exp *heart infarction/ or exp *cardiovascular disease/ or exp *heart disease/ or exp *diabetes mellitus/ or exp *metabolic disorder/ or exp *communicable disease/ or exp *neoplasm/) or exp *Sepsis/ or sepsis.ti,ab.) and (diagnos*.ti,ab. or di.fs.) 2940071

16 13 or 14 or 15 3607334

17 7 and 12 and 16 6917

18 (letter or editorial or comment or book or chapter).pt. 2046551

19 17 not 18 6854

20 limit 19 to english language 6411

21 limit 20 to yr="2002 -Current" 5791

# **PSYCINFO**

| **#** | **Query** | **Limiters/Expanders** | **Last Run Via** | **Results** |
| --- | --- | --- | --- | --- |
| S21 | S4 AND S11 AND S18 | Limiters - Publication Year: 2002-2023 Expanders - Apply equivalent subjects Narrow by Language: - english Search modes - Boolean/Phrase | Interface - EBSCOhost Research Databases Search Screen - Basic Search Database - APA PsycInfo | 2,695 |
| S20 | S4 AND S11 AND S18 | Expanders - Apply equivalent subjects Narrow by Language: - english Search modes - Boolean/Phrase | Interface - EBSCOhost Research Databases Search Screen - Basic Search Database - APA PsycInfo | 3,384 |
| S19 | S4 AND S11 AND S18 | Expanders - Apply equivalent subjects Search modes - Boolean/Phrase | Interface - EBSCOhost Research Databases Search Screen - Basic Search Database - APA PsycInfo | 3,651 |
| S18 | S12 OR S13 OR S17 | Expanders - Apply equivalent subjects Search modes - Boolean/Phrase | Interface - EBSCOhost Research Databases Search Screen - Basic Search Database - APA PsycInfo | 460,363 |
| S17 | S8 AND S16 | Expanders - Apply equivalent subjects Search modes - Boolean/Phrase | Interface - EBSCOhost Research Databases Search Screen - Basic Search Database - APA PsycInfo | 44,198 |
| S16 | S14 OR S15 | Expanders - Apply equivalent subjects Search modes - Boolean/Phrase | Interface - EBSCOhost Research Databases Search Screen - Basic Search Database - APA PsycInfo | 221,238 |
| S15 | (((((DE "Heart Disorders" OR DE "Angina Pectoris" OR DE "Arrhythmias (Heart)" OR DE "Coronary Heart Disease" OR DE "Coronary Thromboses" OR DE "Myocardial Infarctions") OR (DE "Cardiovascular Disorders" OR DE "Aneurysms" OR DE "Arteriosclerosis" OR DE "Blood Pressure Disorders" OR DE "Cerebrovascular Disorders" OR DE "Embolisms" OR DE "Heart Disorders" OR DE "Hemorrhage" OR DE "Hypertension" OR DE "Ischemia" OR DE "Thromboses")) OR (DE "Diabetes" OR DE "Diabetes Insipidus" OR DE "Diabetes Mellitus" OR DE "Type 2 Diabetes")) OR (DE "Metabolism Disorders" OR DE "Cushings Syndrome" OR DE "Cystic Fibrosis" OR DE "Diabetes" OR DE "Hyperglycemia" OR DE "Hypoglycemia" OR DE "Hyponatremia" OR DE "Lipid Metabolism Disorders" OR DE "Phenylketonuria" OR DE "Porphyria")) OR (DE "Infectious Disorders" OR DE "Bacterial Disorders" OR DE "Intracranial Abscesses" OR DE "Parasitic Disorders" OR DE "Sexually Transmitted Diseases" OR DE "Viral Disorders")) OR (DE "Neoplasms" OR DE "Benign Neoplasms" OR DE "Breast Neoplasms" OR DE "Endocrine Neoplasms" OR DE "Leukemias" OR DE "Melanoma" OR DE "Metastasis" OR DE "Nervous System Neoplasms" OR DE "Terminal Cancer") | Expanders - Apply equivalent subjects Search modes - Boolean/Phrase | Interface - EBSCOhost Research Databases Search Screen - Basic Search Database - APA PsycInfo | 144,584 |
| S14 | myocardial infarction or heart attack or cardiovascular disease* or cardiovascular disorder* or diabetes or metabolic disease* or metabolic disorder* or infection disease* or communicable disease* or cancer* or neoplasm* or sepsis | Expanders - Apply equivalent subjects Search modes - Boolean/Phrase | Interface - EBSCOhost Research Databases Search Screen - Basic Search Database - APA PsycInfo | 191,365 |
| S13 | DE "Comorbidity" | Expanders - Apply equivalent subjects Search modes - Boolean/Phrase | Interface - EBSCOhost Research Databases Search Screen - Basic Search Database - APA PsycInfo | 57,720 |
| S12 | ("physical health" or "physical* ill*" or "physical disorder*" or co-occuring or co-occur* or co-morbid* or comorbid* or multimorbid* or multi-morbid* or concomitant or "secondary to medical illness" or “concurrent chronic condition*” or “multiple chronic condition*”) | Expanders - Apply equivalent subjects Search modes - Boolean/Phrase | Interface - EBSCOhost Research Databases Search Screen - Basic Search Database - APA PsycInfo | 428,552 |
| S11 | S5 OR S6 OR S9 OR S10 | Expanders - Apply equivalent subjects Search modes - Boolean/Phrase | Interface - EBSCOhost Research Databases Search Screen - Basic Search Database - APA PsycInfo | 52,273 |
| S10 | ("treatment pattern*" or "treatment gap") | Expanders - Apply equivalent subjects Search modes - Boolean/Phrase | Interface - EBSCOhost Research Databases Search Screen - Basic Search Database - APA PsycInfo | 1,295 |
| S9 | S7 AND S8 | Expanders - Apply equivalent subjects Search modes - Boolean/Phrase | Interface - EBSCOhost Research Databases Search Screen - Basic Search Database - APA PsycInfo | 814 |
| S8 | diagnos* | Expanders - Apply equivalent subjects Search modes - Boolean/Phrase | Interface - EBSCOhost Research Databases Search Screen - Basic Search Database - APA PsycInfo | 421,976 |
| S7 | DE "Errors" OR DE "Error of Measurement" OR DE "Prediction Errors" | Expanders - Apply equivalent subjects Search modes - Boolean/Phrase | Interface - EBSCOhost Research Databases Search Screen - Basic Search Database - APA PsycInfo | 14,070 |
| S6 | "substantial* disadvantag*" | Expanders - Apply equivalent subjects Search modes - Boolean/Phrase | Interface - EBSCOhost Research Databases Search Screen - Basic Search Database - APA PsycInfo | 31 |
| S5 | (overshadow* or (diagnos* n3 (error* or mistake*)) or misdiagnos* or “diagnostic blindspot*” or diagnostic blind spot* or undiagno* or underdiagnos* or (diagnos* n3 (late or delay* or dispari* or inaccura* or oversight* or pitfall*)) or ((complete or incomplete) n3 “physical examination”) or underrecogni* or undertreat* or “under recogni*” or undetect* or (diagnos* n1 gap*) or misinterpret* or mask* or masquerad*) | Expanders - Apply equivalent subjects Search modes - Boolean/Phrase | Interface - EBSCOhost Research Databases Search Screen - Basic Search Database - APA PsycInfo | 50,407 |
| S4 | S1 OR S2 OR S3 | Expanders - Apply equivalent subjects Search modes - Boolean/Phrase | Interface - EBSCOhost Research Databases Search Screen - Basic Search Database - APA PsycInfo | 752,328 |
| S3 | (schizophren* or bipolar*) or delirum or "altered mental status" or ("disease pair analysis" or "symptom-disease pair analysis") or ("anxiety disorder*" or "mood disorder*" or "major depression" or "personality disorder*" or “affective disorder”) | Expanders - Apply equivalent subjects Search modes - Boolean/Phrase | Interface - EBSCOhost Research Databases Search Screen - Basic Search Database - APA PsycInfo | 462,796 |
| S2 | ("Mental disorder*" or "mental* ill*" or "mental health problem*" or psychosis* or (psychiatric n1 (disease* or disorder* illness* or diagnosis* or complaint* or symptom*))) | Expanders - Apply equivalent subjects Search modes - Boolean/Phrase | Interface - EBSCOhost Research Databases Search Screen - Basic Search Database - APA PsycInfo | 309,381 |
| S1 | (((((((DE "Mental Disorders" OR DE "Affective Disorders" OR DE "Anxiety Disorders" OR DE "Autism Spectrum Disorders" OR DE "Bipolar Disorder" OR DE "Borderline States" OR DE "Chronic Mental Illness" OR DE "Dissociative Disorders" OR DE "Eating Disorders" OR DE "Gender Dysphoria" OR DE "Mental Disorders due to General Medical Conditions" OR DE "Neurocognitive Disorders" OR DE "Neurodevelopmental Disorders" OR DE "Neurosis" OR DE "Paraphilias" OR DE "Personality Disorders" OR DE "Psychosis" OR DE "Serious Mental Illness" OR DE "Sleep Wake Disorders" OR DE "Somatoform Disorders" OR DE "Stress and Trauma Related Disorders" OR DE "Substance Related and Addictive Disorders" OR DE "Thought Disturbances") OR (DE "Psychosis" OR DE "Acute Psychosis" OR DE "Affective Psychosis" OR DE "Alcoholic Psychosis" OR DE "Capgras Syndrome" OR DE "Childhood Psychosis" OR DE "Chronic Psychosis" OR DE "Experimental Psychosis" OR DE "Hallucinosis" OR DE "Paranoia (Psychosis)" OR DE "Postpartum Psychosis" OR DE "Reactive Psychosis" OR DE "Schizophrenia" OR DE "Senile Psychosis" OR DE "Toxic Psychoses")) OR (DE "Bipolar Disorder" OR DE "Bipolar I Disorder" OR DE "Bipolar II Disorder" OR DE "Cyclothymic Disorder" OR DE "Mania")) OR (DE "Schizophrenia" OR DE "Acute Schizophrenia" OR DE "Catatonic Schizophrenia" OR DE "Childhood Schizophrenia" OR DE "Paranoid Schizophrenia" OR DE "Process Schizophrenia" OR DE "Schizoaffective Disorder" OR DE "Schizophrenia (Disorganized Type)" OR DE "Schizophreniform Disorder" OR DE "Undifferentiated Schizophrenia")) OR (DE "Anxiety Disorders" OR DE "Castration Anxiety" OR DE "Generalized Anxiety Disorder" OR DE "Obsessive Compulsive Disorder" OR DE "Panic Attack" OR DE "Panic Disorder" OR DE "Phobias" OR DE "Separation Anxiety Disorder" OR DE "Trichotillomania")) OR (DE "Affective Disorders" OR DE "Disruptive Mood Dysregulation Disorder" OR DE "Major Depression" OR DE "Seasonal Affective Disorder")) OR (DE "Major Depression" OR DE "Anaclitic Depression" OR DE "Dysthymic Disorder" OR DE "Endogenous Depression" OR DE "Late Life Depression" OR DE "Postpartum Depression" OR DE "Reactive Depression" OR DE "Recurrent Depression" OR DE "Treatment Resistant Depression")) OR (DE "Personality Disorders" OR DE "Antisocial Personality Disorder" OR DE "Avoidant Personality Disorder" OR DE "Borderline Personality Disorder" OR DE "Dependent Personality Disorder" OR DE "Histrionic Personality Disorder" OR DE "Narcissistic Personality Disorder" OR DE "Obsessive Compulsive Personality Disorder" OR DE "Paranoid Personality Disorder" OR DE "Passive Aggressive Personality Disorder" OR DE "Sadomasochistic Personality" OR DE "Schizoid Personality Disorder" OR DE "Schizotypal Personality Disorder") | Expanders - Apply equivalent subjects Search modes - Boolean/Phrase | Interface - EBSCOhost Research Databases Search Screen - Basic Search Database - APA PsycInfo | 607,995 |

# **CINAHL**

| **#** | **Query** | **Limiters/Expanders** | **Last Run Via** | **Results** |
| --- | --- | --- | --- | --- |
| S20 | S5 AND S13 AND S17 | Limiters - Published Date: 20020101-20221231  Expanders - Apply equivalent subjects  Narrow by Language: - english  Search modes - Boolean/Phrase | Interface - EBSCOhost Research Databases  Search Screen - Advanced Search  Database - CINAHL | 2,785 |
| S19 | S5 AND S13 AND S17 | Expanders - Apply equivalent subjects  Narrow by Language: - english  Search modes - Boolean/Phrase | Interface - EBSCOhost Research Databases  Search Screen - Advanced Search  Database - CINAHL | 2,969 |
| S18 | S5 AND S13 AND S17 | Expanders - Apply equivalent subjects  Search modes - Boolean/Phrase | Interface - EBSCOhost Research Databases  Search Screen - Advanced Search  Database - CINAHL | 2,997 |
| S17 | S14 OR S15 OR S16 | Expanders - Apply equivalent subjects  Search modes - Boolean/Phrase | Interface - EBSCOhost Research Databases  Search Screen - Advanced Search  Database - CINAHL | 1,899,845 |
| S16 | (MH "Myocardial Infarction+") OR (MH "Cardiovascular Diseases+") OR (MH "Diabetes Mellitus+") OR (MH "Metabolic Diseases+") OR (MH "Communicable Diseases+") OR (MH "Neoplasms+") OR (MH "Sepsis+") | Expanders - Apply equivalent subjects  Search modes - Boolean/Phrase | Interface - EBSCOhost Research Databases  Search Screen - Advanced Search  Database - CINAHL | 1,525,584 |
| S15 | myocardial infarction or heart attack or cardiovascular disease* or cardiovascular disorder* or diabetes or metabolic disease* or metabolic disorder* or infection disease* or communicable disease* or cancer* or neoplasm* or sepsis | Expanders - Apply equivalent subjects  Search modes - Boolean/Phrase | Interface - EBSCOhost Research Databases  Search Screen - Advanced Search  Database - CINAHL | 1,155,745 |
| S14 | ("physical health" or "physical* ill*" or "physical disorder*" or co-occuring or co-occur* or co-morbid* or comorbid* or multimorbid* or multi-morbid* or concomitant or "secondary to medical illness" or “concurrent chronic condition*” or “multiple chronic condition*”) | Expanders - Apply equivalent subjects  Search modes - Boolean/Phrase | Interface - EBSCOhost Research Databases  Search Screen - Advanced Search  Database - CINAHL | 201,796 |
| S13 | S6 OR S7 OR S9 OR S10 OR S12 | Expanders - Apply equivalent subjects  Search modes - Boolean/Phrase | Interface - EBSCOhost Research Databases  Search Screen - Advanced Search  Database - CINAHL | 106,167 |
| S12 | S8 AND S11 | Expanders - Apply equivalent subjects  Search modes - Boolean/Phrase | Interface - EBSCOhost Research Databases  Search Screen - Advanced Search  Database - CINAHL | 17,161 |
| S11 | diagnos* | Expanders - Apply equivalent subjects  Search modes - Boolean/Phrase | Interface - EBSCOhost Research Databases  Search Screen - Advanced Search  Database - CINAHL | 1,218,583 |
| S10 | ("treatment pattern*" or "treatment gap") | Expanders - Apply equivalent subjects  Search modes - Boolean/Phrase | Interface - EBSCOhost Research Databases  Search Screen - Advanced Search  Database - CINAHL | 3,115 |
| S9 | (MH "Diagnostic Errors+") | Expanders - Apply equivalent subjects  Search modes - Boolean/Phrase | Interface - EBSCOhost Research Databases  Search Screen - Advanced Search  Database - CINAHL | 21,586 |
| S8 | (MH "Health Care Errors+") OR (MH "Treatment Errors+") | Expanders - Apply equivalent subjects  Search modes - Boolean/Phrase | Interface - EBSCOhost Research Databases  Search Screen - Advanced Search  Database - CINAHL | 49,567 |
| S7 | "substantial* disadvantag*" | Expanders - Apply equivalent subjects  Search modes - Boolean/Phrase | Interface - EBSCOhost Research Databases  Search Screen - Advanced Search  Database - CINAHL | 18 |
| S6 | (overshadow* or (diagnos* n3 (error* or mistake*)) or misdiagnos* or “diagnostic blindspot*” or diagnostic blind spot* or undiagno* or underdiagnos* or (diagnos* n3 (late or delay* or dispari* or inaccura* or oversight* or pitfall*)) or ((complete or incomplete) n3 “physical examination”) or underrecogni* or undertreat* or “under recogni*” or undetect* or (diagnos* n1 gap*) or misinterpret* or mask* or masquerad*) | Expanders - Apply equivalent subjects  Search modes - Boolean/Phrase | Interface - EBSCOhost Research Databases  Search Screen - Advanced Search  Database - CINAHL | 92,232 |
| S5 | S1 OR S2 OR S3 OR S4 | Expanders - Apply equivalent subjects  Search modes - Boolean/Phrase | Interface - EBSCOhost Research Databases  Search Screen - Advanced Search  Database - CINAHL | 676,575 |
| S4 | (MH "Anxiety Disorders+") OR (MH "Depression+") OR (MH "Personality Disorders+") | Expanders - Apply equivalent subjects  Search modes - Boolean/Phrase | Interface - EBSCOhost Research Databases  Search Screen - Advanced Search  Database - CINAHL | 183,349 |
| S3 | (MH "Mental Disorders+") OR (MH "Psychotic Disorders+") OR (MH "Bipolar Disorder+") OR (MH "Schizophrenia+") OR (MH "Affective Disorders+") | Expanders - Apply equivalent subjects  Search modes - Boolean/Phrase | Interface - EBSCOhost Research Databases  Search Screen - Advanced Search  Database - CINAHL | 638,643 |
| S2 | (schizophren* or bipolar*) or delirum or "altered mental status" or ("disease pair analysis" or "symptom-disease pair analysis") or ("anxiety disorder*" or "mood disorder*" or "major depression" or "personality disorder*" or “affective disorder”) | Expanders - Apply equivalent subjects  Search modes - Boolean/Phrase | Interface - EBSCOhost Research Databases  Search Screen - Advanced Search  Database - CINAHL | 160,030 |
| S1 | ("Mental disorder*" or "mental* ill*" or "mental health problem*" or psychosis* or (psychiatric n1 (disease* or disorder* illness* or diagnosis* or complaint* or symptom*))) | Expanders - Apply equivalent subjects  Search modes - Boolean/Phrase | Interface - EBSCOhost Research Databases  Search Screen - Advanced Search  Database - CINAHL | 124,190 |
