# Supplementary material for: Diagnostic inequalities relating to physical healthcare among people with mental health conditions: a systematic review
Source: eClinicalMedicine. 2025 Jan 10;80:103026. doi: 10.1016/j.eclinm.2024.103026 (PMC11773261; doi:10.1016/j.eclinm.2024.103026)
Supplement: Appendix 2 [file mmc2.docx]

**Appendix 2. Studies without a rigorous mental health comparator group**

Like the studies included in the narrative synthesis, studies without a robust comparator group differed in the type of evidence about diagnostic inequalities they offered. Three studies measured diagnostic inequalities that met the definition of diagnostic error, whilst thirty-nine examined patterns of diagnostic inequalities the specific nature of which was not discernible: they may have resulted from patient-related (such as late presentation) and professional-related factors, or both.

All three studies in the diagnostic error group examined the association between altered mental status and missed diagnosis of stroke (Table A). Madsen et (2016) al and Lever et al (2012) reported the rate of individuals with altered mental status who had a missed stroke diagnosis. Because there is no clarity regarding the expected diagnostic error rate, findings are hard to interpret. Venkat et al compared individuals where a stroke diagnosis was missed in the emergency department with those who had stroke diagnosed correctly, and found that the former were more likely to have altered mental status.

The 39 studies in the diagnostic inequalities group (Table B) examined several mental and physical health problems (including cancer, cardiovascular problems, infectious diseases, and sleep-related conditions). Four studies used the general population as a comparator (and were therefore not included in Table 2 studies with a rigorous mental health group). Most studies (n=26) compared the rates of previously known physical illness diagnoses with the rates of detected physical illness at a subsequent, definitive, test. Rates of undiagnosed physical illness ranged from 2.2% to 87.3% of the samples; without indication of the expected under-detection rate, it is difficult to interpret these figures. Studies that used the general population as a comparator group are also reported here.

| **Table A: Diagnostic error** | | | | | | | | | |
| --- | --- | --- | --- | --- | --- | --- | --- | --- | --- |
| **First author, year, DOI** | **Country** | **Study design** | **Data source/ setting** | **Sample size** | **Mental health condition(s). Diagnosis and diagnostic method** | **Physical health condition. Diagnosis and diagnostic method** | **Method of assessment of diagnostic error** | **Comparator group** | **Key outcome(s)** |
|  |  |  |  |  |  |  |  |  |  |
|  |  |  |  |  |  |  |  |  |  |
| **Cardiovascular-related, misdiagnosis** | | | | | | | | | |
| Venkat 2018  10.1159/000491635 | Australia | Retrospective case-control | Emergency department medical records. | 156 misdiagnosed stroke cases (of which n=64 had altered mental status); 156 accurately diagnosed controls ( of which n=40 altered mental status). | Altered mental status, reduced level of consciousness, memory disturbance. | Stroke (excluding Transient Ischemic Attack [TIA]); (ICD-10) | MISSED DIAGNOSIS  Compared patients who received a discharge diagnosis of stroke, but for whom stroke was not diagnosed in ED, with randomly selected patients who had a principal ED and discharge diagnosis of stroke. Checked for ratio of patients with altered mental status in both groups. | Patients with principal ED and discharge diagnosis of stroke, matched for age, gender, language spoken and stroke subtype for the period.  Cases/controls were based on stroke diagnosis (rather than mental status), but the analysis compared altered mental status in the two groups. | Number and % of patients not diagnosed with stroke compared to those who were diagnosed. Altered mental status group: not diagnosed: n=64 (41%); diagnosed: n=40 (26%); p=0.004. |
|  |  |  |  |  |  |  |  |  |  |
|  |  |  |  |  |  |  |  |  |  |
| Madsen 2016  10.1111/acem.13029 | US | Retrospective case-control | Regional database of all strokes reporting all cases of acute ischaemic stroke (AIS) that presented to emergency departments (n=16). | 2027 cases of acute ischemic stroke (AIS) of which n=283 were potentially missed AIS. Of these, n=100 were diagnosed with altered mental status in emergency departments. | Altered mental status (from notes and final diagnoses of emergency department physicians) | Acute ischemic stroke (AIS); (ICD-9, physician verification) | MISSED DIAGNOSIS  Examined all primary and secondary discharge codes to identify cases of AIS. These were compared with presenting diagnoses to determine missed AIS cases in emergency department. Proportion of those initially diagnosed as altered mental status in emergency department was determined. | No comparator group. | 35.3% of all missed cases of AIS had been diagnosed as altered mental status in emergency department. |
|  |  |  |  |  |  |  |  |  |  |
|  |  |  |  |  |  |  |  |  |  |
| **Other physical health conditions, misdiagnosis** | | | | | | | | | |
| Lever 2013  10.1016/j.jen.2012.02.011 | US | Retrospective cohort | Retrospective review of medical records of patients discharged from hospital with ischemic stroke | n=189, of which n=23 with altered mental status | Altered mental status (medical records) | Ischemic stroke (diagnosed by MRI within the first 24 to 48 hours after admission or in a few cases by clinical judgement of the neurology team), | MISSED DIAGNOSIS  Examined association between symptom presentation of patients initially admitted to hospital through the emergency department and later discharged with a diagnosis of ischemic stroke. | No comparison group. | Frequency of altered mental status in patients whose diagnosis of ischemic stroke was missed was 69.6%. (Altered mental status could co-occur with other non-traditional symptoms). |

| **Table B: Diagnostic inequalities** | | | | | | | | | |
| --- | --- | --- | --- | --- | --- | --- | --- | --- | --- |
| **First author, year, DOI** | **Country** | **Study design** | **Data source/setting** | **Sample size** | **Mental health condition(s). Diagnosis and diagnostic method** | **Physical health condition. Diagnosis and diagnostic method** | **Method of assessment of diagnostic inequalities** | **Comparator group** | **Key outcome(s)** |
|  |  |  |  |  |  |  |  |  |  |
|  |  |  |  |  |  |  |  |  |  |
| **Cancer** | | | | | | | | | |
| Attner 2010  10.1159/000315509 | Sweden | Population-based register study; nested case-control study | All patients with cancer diagnoses from 2005 to 2007 were identified in the Cancer Register of Southern Sweden (inpatient and outpatient) | Total case = 167,080. 19,756, control= 147,324. | Dementia | Cancer (gastric, colon, rectal, liver, pancreatic, lung, melanoma, skin cancer, breast, cervical, endometrial, ovarian, prostate, kidney, brain, lymphoma and leukaemia. Only invasive cancers included | UNDERDIAGNOSIS  Investigated the role of dementia on the incidence of cancer for 18 cancer diagnoses. Explored the risk of having a diagnosis of dementia 90 days or more prior to cancer diagnosis, and compared this risk to a cancer-free control group. | Compared between cancer patients and cancer-free patients and looked for incidence of dementia diagnosis. | A diagnosis of dementia was significantly less common among the cancer cases (RR = 0.60, 95% CI = 0.52–0.69). The reduced risk was more pronounced for patients older than 70 years than for patients younger than 70 years (RR = 0.59, 95% CI = 0.52–0.68, vs. RR = 0.73, 95% CI = 0.45–1.19). Authors interpret this as suggesting that cancer is underdiagnosed for persons with dementia. |
| Kisely 2013  10.1001/jamapsychiatry.2013.278 | Australia | Retrospective case-control | Population-based record-linkage analysis | Total new cases of cancer= 135442, of which 6586 occurred in people with mental illness | Psychiatric conditions - ICD9 codes 290-319, and postpartum mental disorders, suicide, and nonaccidental injury | Cancer | LATE-STAGE DIAGNOSIS  Examines proportion of psychiatric patients with metastases at presentation. | Compares between people with psychiatric conditions and the general population. | Proportion of patients with cancer who had metastases at presentation: significantly higher in psychiatric patients (7.1%; 95% CI, 6.5%-7.8%) compared with the general population (6.1%; 95% CI, 6.0%-6.2%). |
| Irwin 2017  10.1634/theoncologist.2016-0489 | US | Retrospective cohort | Medical records of patients with schizophrenia and breast cancer diagnosed between 1993-2015 using the Partners Healthcare System Research Patient Data Registry | Total=95 (of which 32 had screening mammogram documented in the 2 years prior to cancer diagnosis). | Schizophrenia | Breast Cancer | LATE-STAGE DIAGNOSIS  Examined the intervals at each stage from screening to treatment, including (a) positive screening mammogram to biopsy, (b) biopsy to initial treatment (surgery or neoadjuvant chemotherapy) | No comparator group | Median time from positive screening mammogram to biopsy was 1.15 months (interquartile range 0.7–1.95); 24.6% had more than 2 months from screening mammogram to biopsy). |
| Yip 2020  10.1186/s12876-020-01277-0 | Hong Kong, China | Retrospective cohort | Clinical Data Analysis and Reporting System, which represents inpatient and out-patient data of approximately 80% of the local population | 105763 | Psychiatric conditions, including mood disorders, psychotic disorders, drug-induced mental disorders, alcohol induced mental disorders, and other psychiatric illnesses. ICD-9-CM diagnosis codes (290.0–319) | Hepatocellular carcinoma (HCC) and cirrhotic complications | UNDERDIAGNOSIS  Consecutive adult patients in all public hospitals and clinics in Hong Kong with psychiatric diagnoses between year 2003 and 2007, followed for liver-related events (hepatocellular carcinoma (HCC) and cirrhotic complications) and deaths until December 2017. Median follow-up of 12.4 years. | General population for the overall study, but no relevant comparator group is reported for the under-diagnosis data | Compared with the general population, psychiatric patients had increased incidence of HCC (SIR 1.42, 95% confidence interval [CI] 1.28–1.57, P < 0.001). 38.9% of (psychiatric) patients were not known to have liver diseases at the time of liver-related events. |
| Marijanovic 2017  10.24869/psyd.2017.330 | Bosnia and Herzegovina | Cross-sectional | Admission to clinical hospitals | Total=403 (of which 305 were at later stages of disease) | Compares different degrees of depression | Breast cancer (early or late stage presentation to hospital) | LATE-STAGE DIAGNOSIS  Examines depression as a predictor for early or late stage of breast cancer at presentation to hospital. | Compares early stage vs late stage presentation/diagnosis | *There was no statistically significant difference in the degree of depression between women who were diagnosed and treated early and those who were not*. |
| Aggarwal  2008  10.1089/jwh.2007.0544 | US | Prospective cohort | Women’s Health Initiative Observational Study cohort. | With depressive symptoms=12621; no depressive symptoms= 67368 | Depressive symptoms (current and past history of depressive symptoms using the Burnam screen for depression which consists of 6 items from the 20 item CES-D scale and 2 items from the DIS scale) | Breast and colorectal cancer (staged based on Surveillance Epidemiology and End results classification) | LATE-STAGE DIAGNOSIS  Reports odds for later stage of cancer at diagnosis by depressive symptoms. | Uses depressive symptoms as a proxy for mental health diagnosis, and compared between those with and without depressive symptoms | Depressive symptoms were not associated with later stage of either breast or colorectal cancer at diagnosis |
| Dahlman 2022  10.1007/s10552-021-01513-2 | Sweden | Retrospective cohort | Swedish national register | 1,361,532 | Drug use disorders | Prostate cancer | LATE-STAGE DIAGNOSIS  Stage of diagnosis of prostate cancer | Compares between people with psychiatric conditions and the general population. | Men with drug use disorder had higher risk of fatal prostate cancer: HR:1.59 (1.40-1.82, p<0.001). No association was found between drug use disorder and prostate cancer stage at diagnosis. |
| **Diabetes** | | | | | | | | | |
| Foley 2016  10.1093/schbul/sbw027 | Australia | Cross-sectional | Survey of people in contact with public mental health services and non-government organisations. | 356 | Psychosis (ICD-10) | Pre-diabetes and Diabetes Mellitus Type 2 | UNDERDIAGNOSIS  Compares people with previously known diabetes (or pre-diabetes) versus those with newly diagnosed diabetes, identified by the testing in this study, in those with psychotic disorders. | Previously known versus newly diagnosed diabetes/pre-diabetes. | In those with psychotic disorders, just under half of all pre-diabetes or type 2 diabetes diagnosed by this survey (45.9%, 160/356) was already known to participants. |
| Asmelash 2017  10.4314/ejhs.v28i1.2 | Ethiopia | Cross-sectional | University hospital psychiatry clinic | 205 | Major psychiatric problems - including schizophrenia, major depression with psychosis, bipolar with psychosis; participants also had to be taking antipsychotic drugs. | Diabetes Mellitus. Interviews and blood tests to determine diabetes diagnosis | UNDERDIAGNOSIS  Interviews and blood tests to determine diabetes diagnosis, compared with known previous diagnoses of diabetes. | Previously known versus newly diagnosed diabetes. | The prevalence of undiagnosed diabetes was 7.3% (CI: 3.74 - 10.86). Females had 7.1 times the risk of having undiagnosed DM than males (AOR: 7.1, CI: 1.4 - 36.1). Prevalence of undiagnosed diabetes (7.3%) in this study was higher than the estimated national prevalence of Ethiopia - 4.36% reported by the International Diabetes Federation in 2013. |
| Holt 2009  10.1111/j.1464-5491.2009.02742.x | UK | Cross-sectional | Participants from the [Hertfordshire Cohort study](https://generic.wordpress.soton.ac.uk/herts/), data collected by home visit/clinic. | 2997 | Depression | Diabetes | UNDERDIAGNOSIS  Examines number of cases of diagnosed/undiagnosed diabetes in people with depression (measured by HAD-D score [Hospital Anxiety and Depression scale]). | Previously known versus newly diagnosed diabetes. | Of the 37 men and women with HAD-D depression scores of 11 or more, 10 had diabetes, four of whom were previously diagnosed and six of whom were newly diagnosed. Similarly, among the 124 people with HAD-D scores of 8–10, 27 had diabetes, of whom 16 were previously diagnosed and 11 were newly diagnosed. |
| Shafie 2018  10.11622/smedj.2018020 | Singapore | Cross-sectional | Inpatient psychiatric unit | 110 | Schizophrenia, inpatients’ medical records and their current medication use. | Diabetes Mellitus and dyslipidaemia, diagnosis in the medical records, current medications and fasting blood test results. | UNDERDIAGNOSIS  Examines the prevalence and correlates of diabetes and dyslipidaemia, including the incidence of undiagnosed diabetes and dyslipidaemia in a long-stay inpatient schizophrenia population. | Previously known versus newly diagnosed diabetes. | Of the 82.7% (n = 91) of inpatients who were undiagnosed based on medical records and current medications, 2.2% (n = 2) were found to have diabetes mellitus (defined as fasting glucose levels ≥ 7.0 mmol/L). The authors *conclude there are low levels of undiagnosed diabetes, and both diabetes and dyslipidaemia are well-controlled in this population****.*** |
| Taylor 2005  10.1192/bjp.187.5.467 | UK | Cross-sectional | South London and Maudsley NHS trust and Oxleas NHS trust (inpatients) | Total= 606, of which 166 not previously known to have any disorder of glucose homeostasis. | Patients on antipsychotics | Diabetes mellitus. Urinary glucose, fasting plasma glucose, random plasma glucose, glycosylated haemoglobin and oral glucose tolerance tests | UNDERDIAGNOSIS  Examined patients on antipsychotics not known previously to have any disorder of glucose homeostasis. | Previously known versus newly diagnosed diabetes. | Identified 10 cases of impaired fasting glucose and nine cases of diabetes mellitus (11.4% of those tested) among those patients not known previously. |
| **Cardiovascular and metabolic problems** | | | | | | | | | |
| Zavala 2023  10.1192/bjo.2023.12 | Bangladesh/India/Pakistan | Cross-sectional | Adults with SMI attending psychiatric hospitals (inpatients and outpatients) | 3989 (with SMI) | Severe mental illness (SMI) (Schizophrenia, schizotypal and delusional disorders, bipolar affective disorder or severe depression with psychotic symptoms); ICD-10, F20-F29, F30, F31, F32.2, F33.3) | Non-communicable diseases and infectious diseases, including diabetes (HbA1c), hypertension or high blood pressure (blood pressure measurement), hypercholesterolaemia (LDL-cholesterol) | UNDERDIAGNOSIS  Compares new diagnoses of PH conditions in people with SMI with previously known diagnoses. | Previously known versus newly diagnosed. | 94.4% of people with hypercholesterolaemia, 49.2% with diabetes, 48.5% with high blood pressure were previously unaware of their condition (data not reported for other health conditions). |
| Holt 2010  10.1177/0269881109102788 | UK | Cross-sectional | Community and in-patient (for whether screened) / In-patient and out-patient psychiatric units (for prevalence of metabolic syndrome) | Study 1 100 (for whether screened) / Study 2 = main study to look at: 71 (unscreened patients) | Severe mental illness (SMI) (Seven had bipolar illness, 15 schizoaffective disorder, 46 schizophrenia and 3 had unipolar depression) | Metabolic syndrome | UNDERDIAGNOSIS  1) Assess the proportion of people with SMI who had been screened for metabolic abnormalities within the previous year. 2) Assess the prevalence of undiagnosed metabolic abnormalities in people who had not been screened. | Previously known versus newly diagnosed. | Prevalence of undiagnosed metabolic syndrome in people with serious mental illness: n=41/71, 58% had undiagnosed metabolic syndrome. (Components of metabolic syndrome also reported). |
| Larsen 2011 doi.org/10.3109/08039488.2010.486443 | Denmark and Sweden | Cross-sectional | Inpatient and outpatient psychiatric centres | 582 | Schizophrenia | Metabolic syndrome, assessed with laboratory measurements. | UNDERDIAGNOSIS  Assessed proportion of undiagnosed patients (as per medical history), and compares with proportion of diagnosis after screening. | Previously known versus newly diagnosed. | Before screening, 1% of patients were diagnosed with metabolic syndrome. After screening, 43%. |
| Mackin 2007  10.1186/1471-244X-7-28 | UK | Case-control | Psychiatric out-patient clinics in the northeast of England | 106 | Patients on antipsychotic drugs (typical, atypical or combination) and clinically stable. 35.6% had bipolar disorder, 30% schizophrenia, 10% schizo-affective disorder, 24.4 % other mood and anxiety disorders. Case notes and prescription charts | Metabolic syndrome. Blood samples for glucose, HbA1c, insulin and lipid profile. | UNDERDIAGNOSIS  Examines prevalence of untreated metabolic syndrome and baseline and at later follow-up. | Previously known versus newly diagnosed. Compares people on typical and atypical antipsychotics, but not in relation to diagnosis rates. | High prevalence of undiagnosed and untreated metabolic disease at baseline. Six patients had undiagnosed diabetes, six patients had impaired fasting glucose, and eight fulfilled criteria for the metabolic syndrome. Little change between baseline and follow-up despite informing healthcare providers. |
| Player 2008  10.2190/PM.38.1.i | USA | Retrospective cohort | Ambulatory Care settings. National Ambulatory Medical Care Survey and National Hospital Ambulatory Medical Care Survey. | Patient record forms 25,665 and 29,975 PRFs (from the two datasets) | Anxiety (ICD-9-CM) | Unrecognised high blood pressure | UNDERDIAGNOSIS  Assesses how often high blood pressure is unrecognised in ambulatory visits for anxiety, and the factors associated with being unrecognised. | Previously known versus newly recognised high blood pressure, in people presenting with anxiety and hypertension compared to those only with hypertension. | Of visits for anxiety in 2005 with elevated blood pressure, 32.5% were unrecognised. This compares to 24.6% unrecognised for all visits with hypertension/ elevated blood pressure. At visits in which anxiety medications were prescribed (OR 2.44 95%CI 1.01-5.89) and for Hispanic ethnicity (OR 13.9 95%CI 1.46-132.03), high blood pressure was more likely to be unrecognised. |
| Cook 2015  10.1371/journal.pone.0123552 | USA | Cross-sectional | Community mental health program | 457 | Serious mental illness; (DSM-IV-TR) | Common medical comorbidities, including diabetes, hypertension and high cholesterol; (medical examination) | UNDERDIAGNOSIS  Surveys participants to check if they had previously received a diagnosis, and then conducted medical examinations. | Previously known versus newly diagnosed. | 15 individuals had undiagnosed diabetes, 43 with treated but uncontrolled diabetes; 60 individuals had undiagnosed hypertension, and 61 with treated but uncontrolled hypertension; 7 individuals had undiagnosed high cholesterol, and 12 treated but uncontrolled high cholesterol. |
| **HIV** | | | | | | | | | |
| Camoni 2013  10.1093/eurpub/cks122 | Italy | Cross-sectional | Medical records | 3059= late presenters, 2486= non-late presenters | Intravenous drug users (Substance misuse) | HIV. Used individual data on new HIV diagnoses reported in Italy in 2010–2011 to the HIV surveillance system. | LATE-STAGE DIAGNOSIS  Analysed the factors associated with being diagnosed late or at an advanced stage of disease among persons with a new HIV diagnosis in Italy, in the period 2010–2011. | Late vs non-late presenters | Being an intravenous drug user (IDU) was significantly associated with being a late (IDU vs MSM [men who have sex with men], OR = 1.6 [95% CI 1.2-2.1]). The highest proportion of patients diagnosed with advanced HIV were injecting drug users. Suggestive of late diagnosis. |
| Grigoryan 2009  10.1371/journal.pone.0004445 | US | Retrospective cohort | Data from state-wide HIV infection reporting across 33 states | 27572 (cases of HIV infection diagnosed among individuals who inject drugs). | Intravenous drug users (Substance misuse) | HIV | LATE-STAGE DIAGNOSIS  Estimate the proportion of individuals who inject drugs with a late HIV diagnosis (AIDS diagnosis within 12 months of HIV diagnosis) and determine the factors associated with disease progression after HIV diagnosis. | Compares between those who have HIV and inject drugs and those who have HIV and could have been infected in other ways (men who have sex with men [MSM], 39.8%; MSM who also injected drugs, 38.6%; and heterosexual adults at high risk, 36.6%.) | Proportion of individuals who inject drugs who received a late-stage diagnosis of HIV: in 42.2% of individuals who inject drugs, HIV infection progressed to AIDS within 12 months.  A significantly larger proportion of individuals who inject drugs received a late diagnosis compared to other transmission groups (men who have sex with men [MSM], 39.8%; MSM who also inject drugs, 38.6%; and heterosexual adults at high risk, 36.6%) (P=0001; data not shown). |
| Ayano 2020  10.1186/s12879-020-4907-1 | Ethiopia | Cross-sectional | Psychiatric settings | 309 | Severe psychiatric disorders | Chronic viral infections including HIV, hepatitis B virus (HBV) and hepatitis C virus (HCV) | UNDERDIAGNOSIS  Diagnostic patterns assessed by comparing blood samples to previously known diagnosis. | Previously known versus newly diagnosed. | Among patients with chronic viral infections, HIV, HBV and HCV, 76.92, 60, 80, and 75% respectively were undiagnosed. |
| Prevost 2015  10.1111/add.12948 | UK | Cross-sectional | Routine surveillance and survey data | Routine surveillance = 22616; survey data = 2511 | Intravenous drug users (Substance misuse) | Hepatitis C virus (HCV) | UNDERDIAGNOSIS  Information from routine surveillance and survey data was combined to estimate the size of the people who inject drugs population, HCV antibody prevalence and the proportion of HCV antibody prevalent cases who have been diagnosed. | No comparison group. | Proportion of people who have ever injected drugs underdiagnosed with Hepatitis C: 59% [95% CI=43-71%] of prevalent cases. |
| Burton 2019  10.1016/j.jsat.2018.11.008 | USA | Cross-sectional | Urban academically-affiliated medical centre | 597, of which 587 admissions were screened for Hepatitis C Virus | Substance misuse | Hepatitis C Virus (HCV). HCV antibody testing | UNDERDIAGNOSIS  Evaluation of a Hepatitis C Virus (HCV) screening programme for veterans with substance misuse in a substance use treatment programme | Previously known versus newly diagnosed. | 18.9% (14/74) of the HCV-positive cases were newly diagnosed and states they would have likely gone undetected without this program. |
| Pares-Badell 2017  10.1016/j.jsat.2017.03.003 | Spain | Cross-sectional | People who inject drugs who attended the network of state-owned harm reduction centres in Catalonia | 2243 | Intravenous drug users (Substance misuse). Self-report questionnaire responses compared to oral fluid tests. | HIV and Hepatitis C virus (HCV) | UNDERDIAGNOSIS  To estimate the proportion of undiagnosed HIV or Hepatitis C virus (HCV) infection and to assess the risk factors associated with an undiagnosed infection. | Previously known versus newly diagnosed. | 21.5% of all HIV-positive participants were previously undiagnosed and 18.9% of participants who tested positive for HCV were previously undiagnosed. |
| **Sleep-related conditions** | | | | | | | | | |
| McCall 2019  10.1016/B978-0-12-815373-4.00010-1 | US | Cross-sectional (in a sample from an randomised controlled trial) | People being evaluated for participation in an RCT. | 125 | Major depressive disorder (MDD), suicidality and insomnia. | Obstructive sleep apnea (OSA). | UNDERDIAGNOSIS  Examined rates of undiagnosed obstructive sleep apnea in people with suicidality and major depressive disorder. | Previously known versus newly diagnosed. | Proportion of suicidal patients with depression with undiagnosed sleep apnoea: 14% (people with previously diagnosed OSA were excluded). |
| Appleton 2016  10.1016/j.sleh.2017.10.006 | Australia | Cross-sectional | Online survey, community-based sample | 1011 | Depression and/or anxiety. Self-reported in response to survey questions. | Sleep conditions (obstructive sleep apnea (OSA), insomnia, snoring, restless legs). Self-reported in response to survey questions. ICSD-3 criteria. | UNDERDIAGNOSIS  To determine the prevalence of sleep conditions (obstructive sleep apnea (OSA), insomnia, snoring, and restless legs and their relationship with chronic conditions (including depression and anxiety). | Previously known versus newly diagnosed. | Reporting >/= 1 mental health condition (depression and/or anxiety) was independently associated with undiagnosed OSA (OR 6.2 (3.4 to 11.4) |
| Cai 2022 10.1186/s12889-022-12942-2 | China | Cross-sectional | Reviewed existing medical records | Total= 115. OSA=56; Non OSA= 56. | Mild-to-moderate depression or a bipolar disorder. DSM-IV, Hamilton. Depression Rating Scale | Obstructive sleep apnoea. Apnea-hypopnea index (AHI). | UNDERDIAGNOSIS  To investigate clinical risk factors to predict Obstructive Sleep Apnea (OSA) in depression-related conditions. | Comparison is with OSA prevalence in a previous study. | 51.3% of 115 depressive disorder patients had OSA. However previous study reports OSA is present in 11-18% of patients, suggests high rate of undetected OSA in people with Mild-to-moderate depression or bipolar. |
| Hattori 2009  10.1111/j.1440-1819.2009.01956.x | Japan | Cross-sectional | Hospital departments of psychiatry | 32 | Mood disorder, Major depressive disorder, bipolar disorder (Mini-International Neuropsychiatric Interview (MINI); a score of > or =10 on the Hamilton Rating Scale for Depression (HAM-D)) | Obstructive Sleep Apnea Syndrome (OSAS) (definition in study). | UNDERDIAGNOSIS  Compared their diagnosis rate to the previously reported incidence of OSAS in patients with depression. Diagnosed OSAS in mood disorder patients that were previously undiagnosed. | Comparison is with previously reported incidence of OSAS (in different study). | Of the 32 mood disorder patients who met the conditions in the present study, 59.4% had OSAS. All were previously undiagnosed. |
| **Other physical health problems** | | | | | | | | | |
| Ayano 2020  10.1371/journal.pone.0241581 | Ethiopia | Cross-sectional | Specialised mental health hospital | 309 | Severe psychiatric conditions (schizophrenia, bipolar, schizoaffective, and depressive disorders) | Chronic neurologic disorders with episodic manifestations, including epilepsy and migraine headache | UNDERDIAGNOSIS  Logistic regression analysis to examine at the association between diagnosed and undiagnosed cases of neurologic disorders. | Previously known versus newly diagnosed. | Of 309 mental health cases, 14 (4.5%) had undiagnosed neurological disorder, 1% had undiagnosed epilepsy and 3.9% had undiagnosed migraine. Of all cases of neurological disorder (including diagnosed and previously undiagnosed cases), 87.5% were undiagnosed, as were 60% of epilepsy cases and 100% of migraine cases. |
| Aguera-Ortiz 2011  10.1002/gps.2674 | Spain | Cross-sectional | Outpatient psychogeriatric clinic (community mental health) | 99 | Major depressive disorder, bipolar disorder, psychotic disorders, minor depression, anxiety/neurotic disorders, and adjustment disorders; DSM-IV, receiving psychoactive drugs. | Restless leg syndrome (RLS) | UNDERDIAGNOSIS  Re-examination. | Previously known versus newly diagnosed. | Prevalence of restless legs syndrome (RLS) in n=99 psychogeriatric patients: RLS-sure (definite): 11.11%; RLS-pos (possible): 10.1%. None of these patients had received a diagnosis of RLS previously, suggesting underdiagnosis. |
| Koizumi 2013  10.1016/j.genhosppsych.2013.06.007 | Japan | Cross-sectional | Inpatient psychiatric hospitals | 503 | Schizophrenia | Constipation | UNDERDIAGNOSIS  Screened for constipation in people with schizophrenia. If constipation was present, patients were asked if they were aware of it and had reported it to their psychiatrists in charge. | Previously known versus newly diagnosed. | One hundred eighty-four patients with schizophrenia (36.6%) fulfilled the diagnostic criteria for functional constipation. Of these constipated patients, only 103 (56.0%) were aware of it. Only 34 (18.5%) reported constipation to their psychiatrists. |
| Hodgson 2011  10.1097/WAD.0b013e3181f8520a | United States | Cross-sectional | Home visits (community-based) | 265 | Dementia (Alzheimer disease or related). Either a physician diagnosis of dementia, or Mini Mental State Examination (MMSE) score <24. | Acute illness, assessed by Nurse / Laboratory results. | UNDERDIAGNOSIS  Examined prevalence of undiagnosed acute illness | Previously known versus newly diagnosed. | 36% (N=96) of patients had undetected illness. Conditions most prevalent were bacteriuria (15%), hyperglycaemia (6%) and anaemia (5%). |
| Rasanen 2007  doi.org/10.1016/j.eurpsy.2006.09.005 | Finland | Prospective cohort | Long-stay psychiatric inpatient ward | 208 | Schizophrenia (94.7%) or functional psychoses (5.3%). Finnish version of the ICD-9 based on the criteria of the DSM. | Cause of death, hospital treatments due to physical illnesses (based on ICD). | UNDERDIAGNOSIS  To screen for all physical hospitalisations and mortality from physical illnesses in psychiatric patients. | General population | During specialised psychiatric care the majority of the deceased patients had received some somatic treatment for illnesses that ultimately caused their deaths: 81% representing circulatory, 71% digestive, 56% neoplastic, and 36% respiratory ailments. *Authors found no evidence that somatic illnesses in psychiatric patients were underrecognised.* |
| Rokkjaer 2018  10.1080/08039488.2018.1489893 | Denmark | Cross-sectional | Patients living in 4 mental health facilities | 106 | Serious mental illness (SMI): Paranoid schizophrenia, Bipolar affective disorder and Schizoaffective disorder | Somatic disease | UNDERDIAGNOSIS  Determine to what extent previously undiagnosed comorbidities are uncovered when a patient with a mental illness is examined by a general practitioner conducting systematic somatic examinations of psychiatric patients. | Previously known versus newly diagnosed. | Ninety percent of examined patients were found to have previously unknown indications for medical treatment. |
| Rothbard 2009  10.1176/ps.2009.60.4.534 | USA | Cross-sectional | In-patient psychiatric units. Recruited the inpatients who had blood samples available on record. | 588 | Schizophrenia and  major affective disorder | Metabolic and infectious diseases. | UNDERDIAGNOSIS  Determine the rate of previously undiagnosed metabolic and infectious diseases among patients with serious mental illness | Previously known versus newly diagnosed. | A considerable proportion of disease had been missed: (95% of hepatitis B cases, 50% of hepatitis C cases, and 21% of HIV cases) and metabolic disorders (89% of cases with elevated total cholesterol levels and 97% of cases with elevated triglyceride levels). |
| Wong 2015 | Singapore | Cross-sectional | 3 tertiary hospitals | 268 | Dementia. DSM-IV was used for diagnosis, and Clinical Dementia Rating (CDR) was used for assessing severity | Eye pathology: age-related macular degeneration (AMD), diabetic retinopathy (DR), glaucoma, and cataracts. Diagnosed using digital retinal photographs and a questionnaire. | UNDERDIAGNOSIS  Explored the use of a simple retinal photograph to detect four major age-related eye diseases in a cohort of individuals with dementia and the prevalence of eye diseases that are undiagnosed. | Previously known versus newly diagnosed. | 239 (90.5%) had at least one eye disease, 160 of whom (66.9%) had previously undiagnosed conditions. AMD was the most frequent eye condition that was undiagnosed (90.1%), followed by DR (77.6%) and glaucoma (75.7%) |
| Koran 2002  10.1176/appi.ps.53.12.1623 | US | Cross-sectional | Psychiatric inpatient | 289 psychiatric patients | Schizophrenia or other psychotic disorders, mood disorders, adjustment disorders, dementia, organic brain syndrome. | Active and important physical conditions: those that required treatment or continuing medical surveillance, could threaten life or cause or exacerbate a psychiatric disorder. | UNDERDIAGNOSIS  Explored rates of previously undetected conditions. Screened for physical disorders and then medically evaluated if screening suggested positive | Previously known versus newly diagnosed. | % of patients screening positive for physical disorders on admission to a public psychiatric hospital: 29% of patients had physical disorders; of which 24 (20 %) were newly diagnosed for 23 patients (8 %). |
| Woo 2003  10.1177/08919887030160020 | US | Cross-sectional) | Acute geriatric psychiatry unit in a university hospital | 79 | Any (most common were depression and psychosis) | All clinically relevant physical conditions, including constipation, urinary tract infection, hypothyroidism, pneumonia, and several others. | UNDERDIAGNOSIS  Frequency of undiagnosed health problems in a population with mental health problems | Previously known versus newly diagnosed. | Frequency of previously unrecognised medical disorders associated with behavioural disturbances on admission as acute geriatric psychiatry inpatients: At admission, 27 of 79 cases (34%) had unrecognised medical disorders. |
| Feldman 2012  10.4088/JCP.11m07331 | US | Cross-sectional (part of a case-series) | Participants with medical conditions who were excluded from a university trial | 10 people with bipolar disorder | Bipolar disorder | Co-occurring medical conditions | UNDERDIAGNOSIS  People with bipolar disorder found to have certain medical conditions as part of screening for potential participation in a trial, compared with the proportion who had previously been unaware of having those medical conditions | Previously known versus newly diagnosed. | Of 10 people with bipolar who were identified to have certain medical conditions when screened, 7 (70%) had previously been unaware of the medical condition in question |
