# Supplementary material for: Diagnostic inequalities relating to physical healthcare among people with mental health conditions: a systematic review
Source: eClinicalMedicine. 2025 Jan 10;80:103026. doi: 10.1016/j.eclinm.2024.103026 (PMC11773261; doi:10.1016/j.eclinm.2024.103026)
Supplement: Appendix 3 [file mmc3.docx]

**Appendix 3: Risk of bias assessment for Table 1 studies**

| **Newcastle-Ottawa scale** | | | | | | | | |
| --- | --- | --- | --- | --- | --- | --- | --- | --- |
| **Author, year** | **Selection ^a^**  **(max 4 stars)** | | **Comparability ^b^ (maximum 2 stars)** | | | **Outcome/Exposure ^c^ (maximum 3 stars)** | | **Overall RoB** |
| Benitez Majano 2022 | 4* | | 2* | | | 2* | | 8/9 (Low) |
| Mounce 2017 | 3* | | 2* | | | 2* | | 7/9 (Low) |
| Van Hout 2011 | 3* | | 2* | | | 2* | | 7/9 (Low) |
| Walter 2016 | 3* | | 2* | | | 1* | | 6/9 (Moderate) |
| Iglay 2017 | 4* | | 2* | | | 1* | | 7/9 (Low) |
| O'Rourke 2008 | 4* | | 1* | | | 1* | | 6/9 (Moderate) |
| Iachina 2017 | 3* | | 2* | | | 1* | | 6/9 (Moderate) |
| Sharp 2022 | 4* | | 2* | | | 2* | | 8/9 (Low) |
| Waxman 2018 | 4* | | 2* | | | 2* | | 8/9 (Low) |
| Byrd 2012 | 4* | | 2* | | | 2* | | 8/9 (Low) |
| Barin 2020 | 2* | | 2* | | | 1* | | 5/9 (Moderate) |
| Nassery 2021 | 4* | | 0* | | | 2* | | 6/9 (Moderate) |
| Fernholm 2020^d^ | 3* | | 2* | | | 2* | | 7/9 (Low) |
| **RoB 2** | | | | | | | | |
|  | **Randomisation process** | **Deviations from the intended intervention** | | **Missing outcome data** | **Measurement of the outcome** | | **Selection of the reported result** |  |
| Isbell 2023 | Low | Low | | High | Low | | Low | Some |
| McDonald 2003 | Low | Low | | Some | Some | | Some | Some |

^a^ Selection includes: for cohort studies - representativeness of the exposed cohort (1 star max), selection of the non-exposed cohort (1 star max), ascertainment of exposure (1 star max), demonstration that outcome of interest was not present at the start of the study (1 star max); for case-control studies (Fernholm 2020) – is case definition adequate, representativeness of the cases, selection of controls, definition of controls.

^b^ Comparability includes: for both cohort and case-control studies - comparability of cohorts on the basis of the design or analysis (2 stars max).

^c^ Outcome includes: for cohort studies - assessment of outcome (1 star max), was follow-up long enough for outcomes to occur (1 star max), adequacy of follow-up of cohorts (1 star max); Exposure includes - for case-control studies – ascertainment of exposure, same method for ascertainment for cases-controls, non-response rate.

^d^ Case-control study
