# Supplementary material for: Diagnostic inequalities relating to physical healthcare among people with mental health conditions: a systematic review
Source: eClinicalMedicine. 2025 Jan 10;80:103026. doi: 10.1016/j.eclinm.2024.103026 (PMC11773261; doi:10.1016/j.eclinm.2024.103026)
Supplement: Appendix 4 [file mmc4.docx]

**Appendix 4: Risk of bias assessment for Table 2 studies**

| **Newcastle-Ottawa scale (Cohort and case-control studies)** | | | | |
| --- | --- | --- | --- | --- |
| **Author, year** | **Selection ^a^**  **(max 4 stars)** | **Comparability ^b^ (maximum 2 stars)** | **Outcome/Exposure ^c^ (maximum 3 stars)** | **Overall RoB** |
| Fleming 2005 | 4* | 2* | 2* | 8/9 (Low) |
| Goodwin 2014 | 4* | 1* | 2* | 7/9 (Low) |
| Farasatpour 2013 | 3* | 0* | 2* | 5/9 (Moderate) |
| Cunningham 2015 | 4* | 2* | 2* | 8/9 (Low) |
| Cespedes 2020^d^ | 4* | 2* | 2* | 8/9 (Low) |
| O'Rourke 2008 | 4* | 1* | 1* | 6/9 (Moderate) |
| Iritani 2011 | 3* | 1* | 1* | 5/9 (Moderate) |
| Virgilsen 2022 | 4* | 2* | 2* | 8/9 (Low) |
| Chang 2013 | 4* | 2* | 2* | 8/9 (Low) |
| Lin 2016 | 3* | 2* | 2* | 7/9 (Low) |
| Marrie 2009 | 1* | 2* | 0* | 3/9 (High) |
| Guirguis 2017 | 2* | 0* | 1* | 3/9 (High) |
| Heiberg 2019 | 4* | 2* | 2* | 8/9 (Low) |
| Baillargeon 2011 | 4* | 2* | 2* | 8/9 (Low) |
| Gupta 2011 | 4* | 2* | 2* | 8/9 (Low) |
| Puntervold 2021^d^ | 4* | 1* | 2* | 7/9 (Low) |
| Olson 2021 | 3* | 2* | 1* | 6/9 (Moderate) |
| Crump 2013 | 4* | 2* | 2* | 8/9 (Low) |
| **Modified Newcastle-Ottawa scale for cross-sectional studies (scored out of 7)^e^** | | | | |
|  | **Selection (max 3 stars)** | **Comparability (max 2 stars)** | **Outcome (max 2 stars)** | **Overall RoB** |
| Löppönen 2004 | 2* | 1* | 2* | 5/7 (Moderate) |
| Castillo-Sanchez 2018 | 3* | 2* | 2* | 7/7 (Low) |
| Mourinho 2013 | 1* | 1* | 2* | 4/7 (Moderate) |
| Smith 2013 | 3* | 2* | 1* | 6/7 (Moderate) |

^a^ Selection includes: for cohort studies - representativeness of the exposed cohort (1 star max), selection of the non-exposed cohort (1 star max), ascertainment of exposure (1 star max), demonstration that outcome of interest was not present at the start of the study (1 star max); for case-control studies – is case definition adequate, representativeness of the cases, selection of controls, definition of controls.

^b^ Comparability includes: for both cohort and case-control studies - comparability of cohorts on the basis of the design or analysis (2 stars max).

^c^ Outcome includes: for cohort studies - assessment of outcome (1 star max), was follow-up long enough for outcomes to occur (1 star max), adequacy of follow-up of cohorts (1 star max); Exposure includes - for case-control studies – ascertainment of exposure, same method for ascertainment for cases-controls, non-response rate.

^d^ Case-control study

^e^ Cross-sectional study. Modified NOS used for cross-sectional studies. Selection includes - representativeness of the sample (1 star max), non-respondents (1 star max), ascertainment of the exposure (1 star max). Comparability includes – comparability (2 stars max). Outcome includes – assessment of the outcome (1 star max), appropriate statistical text (1 star max).
